# A meta‐analysis of eHealth interventions to promote physical activity in young, middle‐aged, and late middle‐aged adults with obesity or overweight

**DOI:** 10.1111/obr.13898

**Published:** 2025-01-10

**Authors:** Seungmin Lee, Soyeon Ahn, Priya Patel, Nicholas D. Myers

**Affiliations:** ^1^ Department of Kinesiology Iowa State University Ames Iowa USA; ^2^ Department of Educational and Psychological Studies University of Miami Coral Gables Florida USA; ^3^ Department of Kinesiology California State University Fullerton Fullerton California USA; ^4^ Department of Kinesiology Michigan State University East Lansing Michigan USA

**Keywords:** eHealth, exercise, information and communication technology, mHealth

## Abstract

The purpose of this study was to calculate the effects of recent eHealth interventions to promote physical activity in young, middle‐aged, and late middle‐aged adults with obesity or overweight. This meta‐analysis followed the Preferred Reporting Items for Systematic Reviews and Meta‐analyses guidelines. In the search, 3550 articles were identified, and 15 studies met all inclusion criteria. The effects of recent eHealth interventions depended on the type of outcome variable: (a) intensity‐based physical activity (e.g., moderate‐to‐vigorous‐intensity physical activity, average minutes per day from intensity categories, steps per day) or (b) energy expenditure‐based physical activity (e.g., metabolic equivalent of task, kilocalories per week). The overall effects of recent eHealth interventions on the physical activity outcomes in adults with obesity were positive and ranged from small to medium in size. Ethnicity and weight status moderated the effects of recent eHealth interventions on physical activity outcomes. Results from this meta‐analysis provided some evidence for both the utility of, and possible improvements to, eHealth interventions to promote health‐enhancing physical activity in at‐risk populations.

## 1 INTRODUCTION

Physical activity is a modifiable behavior that confers numerous physical and mental health benefits across populations. Physically active individuals tend to perform better in various cognitive areas such as attention, processing speed, executive function, and memory, compared to those who are inactive.^1^ Those who are physically active also experience improvements in sleep, like falling asleep faster, and often report enhanced well‐being.^2^, ^3^, ^4^ Additionally, research shows that higher levels of physical activity are linked to a lower risk of developing certain cancers, type 2 diabetes, and reduced blood pressure.^5^, ^6^, ^7^ As noted in the Physical Activity Guidelines Advisory Committee Scientific Report,^8^, ^9^ even a single session of moderate‐to‐vigorous physical activity can offer some of the mentioned benefits. With consistent participation in such activity, these benefits tend to increase over time.

Physical activity can be just as crucial, if not more so, for young, middle‐aged, and late middle‐aged adults with obesity or overweight. For textual (e.g., word limits) and conceptual (e.g., eligibility) simplicity, from this point forward, we will refer to this population as simply “adults with obesity.” Use of this expression is in alignment with national physical activity guidelines where the overweight classification often is viewed as a “pre‐obesity” classification from an intervention perspective (e.g., persons with overweight often are eligible for interventions for persons with obesity).^8^ We acknowledge that some scholars may prefer the more precise term, “adults with obesity or overweight,” as it can be more accurate from a body fat categorization perspective, though it may be less unifying and more cumbersome for intervention eligibility. Physical activity has consistently been recommended for individuals with obesity due to its independent effect on health‐related outcomes, regardless of body weight.^10^, ^11^, ^12^ This indicates that sufficient levels of physical activity may mitigate the negative effects of excess body weight on health outcomes. Adults with obesity can significantly benefit from regular physical activity, even in the absence of weight loss, for several reasons. For example, adults with obesity may experience: (a) improvements in blood pressure, insulin sensitivity, and body composition; (b) relative reduction in incidence or progression of a chronic disease; and (c) prevention of weight gain, by promoting their physical activity regardless of their weight change.^8^, ^9^ Thus, physical activity is an excellent intervention target for adults with obesity to improve their health, whether or not weight loss is achieved.

Unfortunately, there is evidence that adults with obesity tend to be physically active only when trying to lose weight.^13^ Promoting physical activity only for weight loss can be problematic because adults with obesity may lose motivation for physical activity if weight loss is not achieved at a satisfactory rate, or not at all. Perhaps relatedly, there have been persistent reports that most adults with obesity do not meet public health guidelines for physical activity,^14^, ^15^ for example, 150 min per week of moderate physical activity.^8^ Therefore, developing and testing behavioral interventions that are designed to promote physical activity in adults with obesity, beyond possible weight loss, are important because of the potential health benefits of physical activity experienced by adults with obesity—even without weight loss.

As the technical capacity and accessibility of the Internet via electronic and mobile devices increase, it becomes feasible to conduct eHealth intervention research in real‐world settings. In this study, eHealth is defined broadly as the use of information and communication technology (e.g., computer, smartphone) to improve health or enable health care, consistent with previous literature.^16^, ^17^, ^18^ Compared to a typical intervention (e.g., face‐to‐face), eHealth interventions can offer multiple potential advantages^17^, ^19^: (a) access to information and support on demand, (b) possibility for users to remain anonymous while seeking information and support regarding a sensitive and private health issue, (c) ability to maintain or update behavioral change techniques based on current scientific knowledge, and (d) capability of combining means of communication to address a particular purpose of an intervention. We note that the definition of eHealth can vary because it has been applied in various contexts, and the advancements of information and communication technologies are ongoing.

This meta‐analysis built off a previous systematic review that did not include any quantitative effect size calculation on the effects of recent eHealth interventions to promote physical activity in adults with obesity.^20^ To our knowledge, no studies have calculated the effects of recent eHealth intervention research focusing on “promoting physical activity” in “adults with obesity.” To be specific, there are review studies on eHealth interventions to promote physical activity but in other populations, instead of adults with obesity, for example, any adults,^21^ adults with some cancers,^22^, ^23^ adults with type 2 diabetes who are not necessarily with obesity,^24^ working‐age women,^18^ and older adults.^25^, ^26^ There are review studies on eHealth interventions for weight loss or weight maintenance in adults with obesity,^27^, ^28^, ^29^, ^30^ but not focusing on physical activity as an outcome. We believe that it is important to separate efforts that are exclusive to physical activity because this behavior can yield multiple health benefits (e.g., reducing the risk of developing a new chronic disease) even without weight loss. Recently, some review studies with meta‐analyses investigated effective behavioral change techniques (i.e., intervention components) for physical activity in adults with obesity,^31^, ^32^, ^33^ but did not focus on empirically synthesizing effects of eHealth interventions (i.e., physical activity promotion).

The purpose of this study was to calculate the effects of recent eHealth interventions to promote physical activity in adults with obesity. Two research questions were investigated.

1. What are the overall effects of recent eHealth interventions on physical activity outcomes, compared to control groups?
2. Do the effects of recent eHealth interventions on physical activity outcomes vary based on different moderators?

## 2 METHOD

This study followed the Preferred Reporting Items for Systematic Reviews and Meta‐analyses guidelines.^34^ Accordingly, we clearly defined the inclusion and exclusion criteria and provided a rationale to ensure clarity and transparency in the reporting of this meta‐analysis. This meta‐analysis built off a recent systematic review (without any quantitative effect size calculation) on eHealth physical activity interventions for adults with obesity.^20^ In the recent systematic review, 18 out of 2276 articles published from 2010 to 2020 were qualitatively summarized. In this meta‐analysis, 15 out of 3550 articles published from 2010 to 2024 were included to synthesize the intervention effects using a quantitative meta‐analytic technique. Some qualitative information (e.g., characteristics of interventions) from seven studies^35^, ^36^, ^37^, ^38^, ^39^, ^40^, ^41^ in this meta‐analysis was previously reported in the aforementioned systematic review. The effect sizes reported in this meta‐analysis have not been used for any publication.

### 2.1 Search strategy

#### 2.1.1 Eligibility criteria

There were seven inclusion criteria in this meta‐analysis. First, studies should be peer‐reviewed and full‐text articles published between 2010 and 2024 in English. Second, studies should investigate the use of eHealth interventions to promote physical activity. In this meta‐analysis, eHealth interventions encompassed various forms of information and communication technology (e.g., website, mobile phone application, text messaging), consistent with the previous literature.^8^, ^16^ Third, studies must be a randomized controlled trial with both a treatment group and a control group (e.g., waitlist group, usual care group) to test the effect of eHealth. Fourth, studies should focus on adults aged 18 to 65, specifically young, midlife, and late midlife adults with obesity and overweight, collectively referred to as “adults with obesity” in this study. This criterion aligns with national physical activity guidelines, particularly the 2018 Physical Activity Guidelines Advisory Committee Scientific Report, which categorizes populations by age.^8^ The upper limit of 65 years is based on the standard retirement age in the United States and several European countries. Although emerging evidence suggests that eHealth interventions, especially those utilizing wearable activity tracker‐based programs, can effectively promote physical activity among older adults,^25^, ^42^, ^43^, ^44^ assessing the effect size specifically for older adults with obesity and overweight is beyond the scope of this study. By focusing on non‐older adults with obesity, a meta‐analysis may statistically assess outcomes relevant to the focal population, without the confounding effects of combining age groups with differing needs or responses.^45^ Fifth, studies must target adults who are considered at least overweight, meaning that eligible studies should include those with a body mass index [BMI] of 25.00 kg/m^2^ or higher. The rationale for this eligibility is that physical activity can provide benefits to adults with excess weight, whether categorized as overweight or obese, who may encounter elevated risks of various health issues (e.g., type 2 diabetes mellitus, hypertension, cardiovascular diseases).^46^ This eligibility criterion was consistent with previous reviews of physical activity interventions (often with in‐person delivery) for adults with obesity.^20^, ^47^, ^48^ Sixth, studies must use a physical activity assessment (e.g., device‐based or questionnaire‐based) as a continuous outcome measure (e.g., moderate‐to‐vigorous‐intensity physical activity [MVPA], metabolic equivalent of task [MET]) that was clearly reported in the results section of the articles. Seventh, studies should provide sufficient statistical information to obtain or calculate an effect size and its variance. There were two exclusion criteria in this study. First, feasibility or pilot studies were excluded because they may not have sufficient power. According to the Consolidated Standards of Reporting Trials (CONSORT) statement,^49^, ^50^, ^51^ a feasibility study assesses whether a proposed project can be implemented, evaluates its suitability, and identifies the most effective approach for execution prior to a full‐scale launch. A pilot study answers similar questions but goes a step further by running a small version of the future study, or part of it, to test if the study's design and procedures work well before the full‐scale launch. Pilot studies fall under the broader category of feasibility studies, rather than being distinct or mutually exclusive. Feasibility or pilot studies typically do not include a sample size calculation, as they aim to gather preliminary data (e.g., recruitment and participation rates) rather than assess efficacy or effectiveness.^49^, ^50^, ^51^ Including such studies in a meta‐analysis could potentially bias the results, particularly if they lack statistical power or are not directly aligned with the objectives of the meta‐analysis (e.g., assessing eHealth effects). We screened feasibility and pilot studies by examining explicit indicators such as study titles. Second, gray literature was excluded in this study because of some potential limitations such as limited access and interim data.^52^

#### 2.1.2 Databases and search keywords

Five electronic databases were used: CINAHL, Cochrane, Embase, PsycINFO, and PubMed, based on optimal database combinations for physical activity literature searches in review studies.^53^, ^54^ A literature search from 2010 to 2024 was conducted consistent with recent review studies on eHealth interventions to promote physical activity in other populations.^23^, ^26^ Search keywords for relevant scope (e.g., physical activity, eHealth interventions, adults with obesity) in the databases were developed and revised as needed by the first author and a librarian from a research university. The search was conducted on 29 October 2024. De‐duplication from the databases was conducted through a reference management software. Full information about search keywords and search results from each database is provided in Table S1.

### 2.2 Screening strategy

#### 2.2.1 Study selection

The first author screened the searched studies, and the third author independently screened ~30% of the total number of the studies to balance efficiency and rigor. There were two steps of screening for study selection. In the first step, screening titles and abstracts was conducted to exclude studies that were obviously not relevant. In the second step, full texts of potentially relevant articles were examined against the aforementioned eligibility criteria. Before initiating each step, calibration exercises (i.e., piloting the screening on several randomly selected studies) were performed by the first and third author. Interrater reliability for study selection was measured by percent of agreement. Any disagreements in the study selection were discussed and resolved by the two authors. Interrater reliability for the first step of screening was 97%. Interrater reliability for the second step of screening was also 97%.

### 2.3 Data collection strategy

#### 2.3.1 Risk of bias

After the study selection, risk of bias in each of the included studies was evaluated independently by the first and third author. The Cochrane Risk‐of‐Bias tool Version 2 (RoB 2) was used.^55^ The RoB 2 tool consists of five domains (e.g., Domain 1: risk of bias arising from the randomization process). Based on the domains, the overall risk of bias for each study was judged as low, some, or high concerns. Similar with the study selection process, calibration exercises were performed by the two authors. Interrater reliability for the evaluation of risk of bias was measured by percent of agreement. Any disagreements in the evaluation were discussed and resolved by the two authors. Interrater reliability ranged from 85% (e.g., Domain 3: risk of bias due to missing outcome data) to 100% (e.g., Domain 1: risk of bias arising from the randomization process). The risk‐of‐bias assessment figures were made by using robvis.^56^

#### 2.3.2 Data extraction

Characteristics of the study designs and interventions, physical activity assessment, and statistical information (e.g., *M, SD, n* of physical activity assessment in each group at pretest or posttest) in each study were recorded using a coding scheme. The coding scheme was developed by the first author under the guidance of the second author who is a meta‐analyst. For example, in the coding scheme, a variety of physical activity measurements were classified by either intensity‐based physical activity or energy expenditure‐based physical activity, consistent with a guideline of physical activity assessment.^57^ Intensity‐based physical activity was a type of outcome variable assessed by computing how much a person performs different physical activity intensity categories (e.g., MVPA, average minutes per day from intensity categories, steps per day). Intensity‐based physical activity reflects the focus on how much physical activity is performed, with attention to intensity categories. Energy expenditure‐based physical activity was a type of outcome variable assessed by determining the energy expenditure in the physical activity (e.g., [MET], kilocalories per week). Energy expenditure‐based physical activity reflects the focus on caloric or metabolic output. After developing the coding scheme, each of the included studies was independently coded by the first and third author. Interrater reliability for the data extraction was measured by percent of agreement. Any disagreements in the evaluation were discussed and resolved by the two authors. Interrater reliability ranged from 85% (e.g., *n* in control group at posttest) to 100% (e.g., *M* of intensity‐based physical activity in control group at pretest).

### 2.4 Data synthesis

#### 2.4.1 Effect size

To calculate the intervention effect, we computed the difference between the mean outcome values of the intervention and control groups in the posttest and pretest measures. This difference represents the change in each outcome between two groups, indicating the intervention gain or loss. Specifically, we calculated unbiased estimates of the effect sizes (*g*_*ppc*_) for each outcome and their associated variance,^58^ incorporating the imputed correlation value of 0.5 between the pre‐ and posttest scores.

The unbiased (*g*_*ppc*_) estimate was computed using an equation^58^:

$$g_{\mathrm{ppc}}=\left(1-\frac{3}{4\left(n_{E}+n_{C}-2\right)-1}\right)\left[\frac{\left(M_{\text{post},E}-M_{\mathrm{pre},E}\right)-\left(M_{\text{post},C}-M_{\mathrm{pre},C}\right)}{{\mathrm{SD}}_{\mathrm{pre}}}\right],$$

where *M* is mean; *SD* is standard deviation; *n* is sample size; *E* is experimental group; *C* is control group; post is posttest score; pre is pretest score. Its associated variance is

$$\sigma_{g_{\mathrm{ppc}}}^{2}=2{\left(1-\frac{3}{4\left(n_{E}+n_{C}-2\right)-1}\right)}^{2}\left(1-r\right)\left(\frac{n_{E}+n_{C}}{n_{E}n_{C}}\right)\left(\frac{n_{E}+n_{C}-2}{n_{E}+n_{C}-4}\right)\left(1+\frac{{g_{\mathrm{ppc}}}^{2}}{2\left(1-r\right)\left(\frac{n_{E}+n_{C}}{n_{E}n_{C}}\right)}\right)-{g_{\mathrm{ppc}}}^{2},$$

where *r* is 0.5. In accordance with guidelines for interpreting effect sizes,^59^ common heuristics were employed to facilitate the interpretation of the absolute value of g_ppc_: 0.20 is considered a small effect, 0.50 a medium effect, and 0.80 a large effect.

#### 2.4.2 Publication bias

We evaluated the potential presence of publication bias. Publication bias occurs due to the tendency that studies with a significant effect in a favorable direction are more likely to be published. To assess this bias, we employed various indicators: (a) Begg and Mazumdar's rank correlation test for detecting asymmetry in funnel plot, (b) Egger's regression test of intercept, and (c) examination of funnel plots for asymmetry. If the null hypothesis, which assumes no relationship between effect sizes and their associated variance, is rejected, it indicates substantial evidence in favor of publication bias.

#### 2.4.3 Handling dependencies in effect size estimates

Since several studies employed multiple outcomes (*k* = 9) and included more than two comparison groups (*k* = 3), multiple effect sizes were extracted from these studies, thereby violating the assumption of independence.^60^ The dependency issues were addressed by using several methods.^45^ First, we computed the overall intervention effects and analyzed each type of outcome variables. Then, in cases where dependencies still persisted within each outcome type, the dependent effect sizes and their associated standard errors were averaged.

#### 2.4.4 Statistical analyses

The statistical analyses were based on relevant statistical methods for meta‐analysis.^61^, ^62^ All analyses were performed using *metafor* package in R. Initially, under the fixed‐effects model, the effect sizes were weighted by the inverse of their variance. An overall homogeneity test of these effects (*Q*_*total*_) was performed. If the fixed‐effects model was not supported, the random‐effects model was applied. The random‐effects model incorporated the additional between‐study variability, which was estimated using the Restricted Maximum Likelihood Estimation. Additionally, the mixed‐effects model was applied. The mixed‐effects model incorporated the uncertainty within each study‐level predictor (e.g., gender, risk of bias), with weights computed for each level of moderators. More details about a random‐effects or mixed‐effects model with a categorical moderator can be found elsewhere.^63^

## 3 RESULTS

### 3.1 Study selection

**FIGURE 1:**
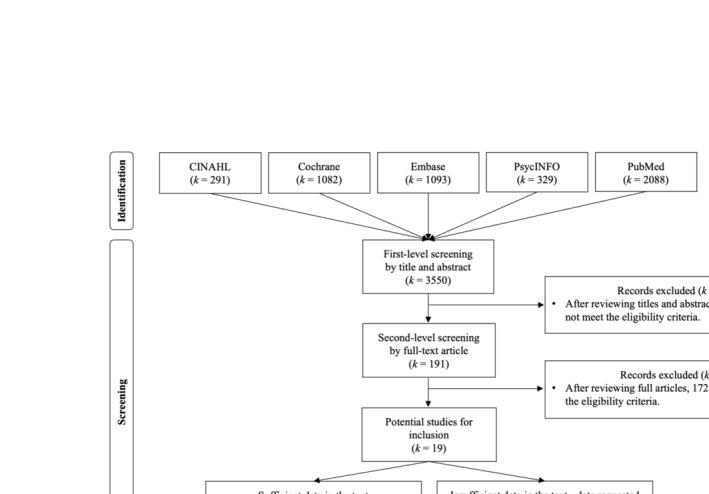
Flowchart of study inclusion process.

The flowchart of study inclusion process is provided in Figure 1. A total of 3550 articles were identified using the search terms from the five databases. After the first‐step screening of titles and abstracts of these articles, 191 articles remained. In the screening, studies were excluded due to obvious reasons (e.g., observational studies, participants who are less than 18 years old). After the second‐step screening of the full‐text versions of these articles, 19 articles remained. Each eligibility and exclusion criterion led to the exclusion of the articles, with the age criterion accounting for the highest rate of exclusions (59%) and gray literature the lowest (1%). The reasons for excluding each study during the second‐step full‐text screening of retrieved articles are fully provided in Table S2 (i.e., 0, *ineligible*; 1, *eligible for each criterion*). The 19 remaining studies were considered as potential studies for inclusion and were further classified as providing sufficient data or providing insufficient data within the published manuscript. Thirteen studies were classified as providing sufficient data. Six studies were classified as providing insufficient data. The first author of this manuscript contacted the corresponding author of each study with insufficient data and requested information for effect size computation. Authors from two studies with insufficient data provided additional data for effect size computation. As a result, 15 studies were included in this meta‐analysis.

### 3.2 Characteristics of study designs and eHealth interventions

The characteristics of study designs and eHealth interventions are described in Table 1. The included studies in this meta‐analysis were conducted across the world including Australia, France, Iran, Ireland, Japan, the Netherlands, Spain, and the United States. The number of sample sizes ranged from 39 to 650 across the studies. From these 15 independent peer‐reviewed published studies, a total of 18 effect sizes were extracted. The total sample size across all studies comprised 3121 participants (*M* = 208.07, *SD* = 218.34, *Min* = 39, *Max* = 650), including 985 males (*M* = 72, *SD* = 129, *Min* = 0, *Max* = 442) and 2031 females (*M* = 149, *SD* = 185, *Min* = 0, *Max* = 565). The average age of participants was 44.66 years (*SD* = 6.18, *Min* = 32.50, *Max* = 56). Most of the studies (*k* = 13, 87%) used non‐probability sampling. The majority of studies (*k* = 12, 80%) recruited adults with at least overweight (i.e., ≥25.00 kg/m^2^), and other studies only recruited either adults with overweight (*k* = 1, 7%) or adults with obesity (*k* = 2, 13%). While approximately half of the studies did not report the ethnic backgrounds of participants (*k* = 8), one study focused on African American participants, and the remaining studies included participants from multiple ethnic backgrounds. The majority of studies (*k* = 13, 87%) used individual‐level randomization, and two studies (13%) used cluster‐level randomization. Four studies (27%) employed participant matching based on individual backgrounds (e.g., BMI, gender, city).

**TABLE 1:** Study design and eHealth interventions in included studies for meta‐analysis.

Study information	Study design	Physical activity assessment	eHealth intervention
Study 1 Ainscough et al., 2019 ^35^ Country: Ireland	N: 565 Sampling: non‐probability BMI: 25 or higher Gender: female only Ethnicity: multiple ethnic Assignment method: individual Matching: BMI	Type: questionnaire‐based Measurement: MET‐mins/week (Energy‐based PA), moderate PA mins/week (Intensity‐based PA)	Purpose: health‐enhancing behavior Theory use: social cognitive theory Technology: mobile phone‐based Intervention Duration: NR
Study 2 Duncan et al., 2020 ^64^ Country: Australia	N: 116 Sampling: non‐probability BMI: 25 or higher Gender: female, male Ethnicity: NR Assignment method: individual Matching: BMI	Type: device‐based, questionnaire‐based Measurement: MVPA mins/week (Intensity‐based PA), MVPA mins/day (Intensity‐based PA)	Purpose: weight loss or maintenance Theory use: social cognitive theory Technology: mobile phone‐based Intervention duration: 6–12 months
Study 3 van Genugten et al., 2012 ^65^ Country: Netherlands	N: 539 Sampling: non‐probability BMI: 25 or higher Gender: female, male Ethnicity: NR Assignment method: individual Matching: sex	Type: questionnaire‐based Measurement: average PA mins/day from PA intensity categories (Intensity‐based PA)	Purpose: weight loss or maintenance Theory use: self‐regulation theory Technology: web‐based Intervention duration: less than 6 months
Study 4 Lison et al., 2020 ^66^ Country: Spain	N: 105 Sampling: non‐probability BMI: 25.00–29.99 Gender: NR Ethnicity: NR Assignment method: individual Matching: NR	Type: device‐based Measurement: average count per minute (Energy‐based PA)	Purpose: health‐enhancing behavior Theory use: NR Technology: web‐based Intervention duration: less than 6 months
Study 5 Nazari et al., 2020 ^36^ Country: Iran	N: 91 Sampling: non‐probability BMI: 25 or higher Gender: female only Ethnicity: NR Assignment method: cluster Matching: NR	Type: questionnaire‐based Measurement: MET/week (Energy‐based PA)	Purpose: health‐enhancing behavior Theory use: social cognitive theory Technology: web‐based Intervention duration: less than 6 months
Study 6 Nakata et al., 2019 ^37^ Country: Japan	N: 95 Sampling: non‐probability BMI: 25 or higher Gender: female, male Ethnicity: NR Assignment method: individual Matching: sex, city	Type: device‐based Measurement: steps/day (Intensity‐based PA), MVPA mins/day (Intensity‐based PA)	Purpose: weight loss or maintenance Theory use: NR Technology: activity monitor‐based Intervention duration: more than 12 months
Study 7 Patrick et al., 2011 ^38^ Country: NR	N: 441 Sampling: non‐probability BMI: 25 or higher Gender: male only Ethnicity: multiple ethnic Assignment method: individual Matching: NR	Type: questionnaire‐based Measurement: light walking‐mins/day (Intensity‐based PA), MET‐mins/week for other PA (Energy‐based PA)	Purpose: weight loss or maintenance Theory use: social cognitive theory Technology: web‐based Intervention duration: 6–12 months
Study 8 Pellegrini et al., 2012 ^39^ Country: NR	N: 51 Sampling: non‐probability Sampling: NR BMI: 25 or higher Gender: female, male Ethnicity: multiple ethnic Assignment method: individual Matching: NR	Type: questionnaire‐based Measurement: kcal/week (Energy‐based PA)	Purpose: weight loss or maintenance Theory use: NR Technology: activity monitor‐based Intervention duration: 6–12 months
Study 9 Rogers et al., 2016 ^40^ Country: NR	N: 39 Sampling: non‐probability BMI: 30 or higher Gender: female, male Ethnicity: multiple ethnic Assignment method: individual Matching: NR	Type: questionnaire‐based Measurement: kcal/week (Energy‐based PA)	Purpose: weight loss or maintenance Theory use: NR Technology: activity monitor‐based Intervention duration: 6–12 months
Study 10 Steinberg et al., 2013 ^41^ Country: USA	N: 91 Sampling: non‐probability BMI: 25 or higher Gender: female, male Ethnicity: multiple ethnic Assignment method: individual Matching: NR	Type: questionnaire‐based Measurement: kcal/week (Energy‐based PA)	Purpose: weight loss or maintenance Theory use: self‐regulation theory Technology: web‐based Intervention duration: 6–12 months
Study 11 Lugones‐Sanchez et al., 2022 ^67^ Country: Spain	N: 650 Sampling: probability BMI: 25 or higher Gender: female, male Ethnicity: NR Assignment method: individual Matching: NR	Type: questionnaire‐based Measurement: MVPA mins/week (Intensity‐based PA), MET‐mins/week (Energy‐based PA)	Purpose: health‐enhancing behavior Theory use: NR Technology: activity monitor‐based Intervention duration: less than 6 months
Study 12 Bughin et al., 2021 ^68^ Country: France	N: 50 Sampling: non‐probability BMI: 30 or higher Gender: female, male Ethnicity: NR Assignment method: individual Matching: NR	Type: device‐based Measurement: MVPA mins/week (Intensity‐based PA), steps/day (Intensity‐based PA)	Purpose: health‐enhancing behavior Theory use: NR Technology: mobile phone‐based Intervention duration: less than 6 months
Study 13 Gerber et al., 2013^69^ Country: NR	N: 88 Sampling: non‐probability BMI: 25 or higher Gender: female only Ethnicity: African American only Assignment method: cluster Matching: NR	Type: questionnaire‐based Measurement: MVPA mins/day (Intensity‐based PA), moderate PA mins/day (Intensity‐based PA)	Purpose: weight loss or maintenance Theory use: NR Technology: web‐based Intervention duration: 6–12 months
Study 14 Black & Brunet, 2021 ^70^ Country: NR	N: 47 Sampling: non‐probability BMI: 25 or higher Gender: female only Ethnicity: multiple ethnic Assignment method: individual Matching: NR	Type: questionnaire‐based Measurement: MET‐mins/week for walking (Energy‐based PA), MET‐mins/week for other PA (Energy‐based PA)	Purpose: health‐enhancing behavior Theory use: self‐determination theory Technology: activity monitor‐based Intervention duration: less than 6 months
Study 15 Kohl et al., 2023^71^ Country: NR	N: 153 Sampling: non‐probability BMI: 25 or higher Gender: female, male Ethnicity: NR Assignment method: individual Matching: NR	Type: device‐based, questionnaire‐based Measurement: MVPA mins/week (Intensity‐based PA), MVPA mins/week (Intensity‐based PA)	Purpose: health‐enhancing behavior Theory use: NR Technology: web‐based Intervention duration: less than 6 months

Abbreviations: BMI, body mass index; MET, metabolic equivalent of task; MVPA, moderate‐to‐vigorous physical activity; NR, not reported; PA, physical activity.

Regarding physical activity assessment, more than half of the included studies (*k* = 10, 67%) used questionnaire‐based assessment (e.g., self‐report, structured interview), and the other studies (*k* = 5, 33%) used device‐based assessment (e.g., accelerometer). Approximately half of the included studies (*k* = 8, 53%) provided more than one physical activity measurement (e.g., MET‐mins/week + moderate physical activity mins/week). In total, there were 24 physical activity measurements across the included studies (see Table 1), which resulted in 18 physical activity outcomes that were either intensity‐based physical activity (*k*_*effect*_ = 9) or energy expenditure‐based physical activity (*k*_*effect*_ = 9).

All of the interventions from the included studies intended to promote physical activity in adults with obesity; however, they seemed developed for a specific purpose, which can be categorized into two categories: weight loss or maintenance (*k* = 8, 53%) and health‐enhancing behavior (*k* = 7, 47%). Less than half of the included studies (*k* = 7, 47%) explicitly reported the use of theory to develop their interventions: social cognitive theory (*k* = 4), self‐regulation theory (*k* = 2), or self‐determination theory (*k* = 1). A variety of intervention technology was employed in the included studies and can be categorized by web‐based (*k* = 7, 47%), mobile phone‐based (*k* = 3, 20%), and physical activity monitor‐based (*k* = 5, 33%). To be specific, a web‐based intervention was typically delivered through a website. A mobile phone‐based intervention was delivered through mobile application or text message. A physical activity monitor‐based intervention was provided with a commercial‐graded accelerometer combined with a small screen, email, website, or mobile application using the Internet. A number of different intervention durations were employed and can be categorized by <6 months (*k* = 7, 47%); 6–12 months (*k* = 6, 40%); >12 months (*k* = 1, 7%); and not reported (*k* = 1, 7%). The characteristic summary of included studies is provided in Table S3.

### 3.3 Risk of bias

**FIGURE 2:**
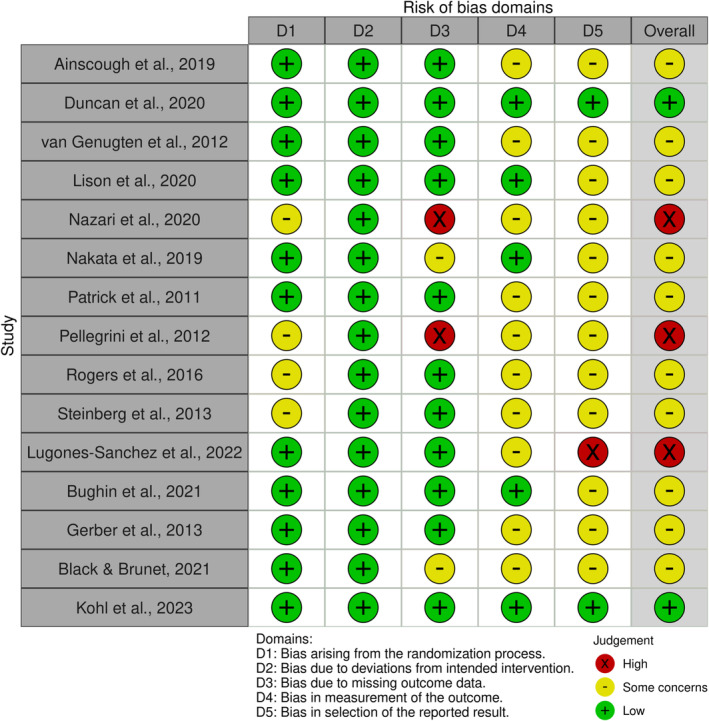
The evaluation of risk of bias.

The evaluation of risk of bias is provided in Figure 2. Regarding the overall risk of bias based on RoB 2, the included studies had low concerns (*k* = 2, 13%), some concerns (*k* = 10, 67%), and high concerns (*k* = 3, 20%). To be specific, low risk ranged from 13% (*k* = 2) in Domain 5 (i.e., bias in selection of the reported result) to 100% (*k* = 15) in Domain 2 (i.e., bias due to deviations from intended intervention). Some concerns ranged from 0% (*k* = 0) in Domain 2 to 80% (*k* = 12) in Domain 5. High risk ranged from 0% (*k* = 0) in Domain 2 to 13% (*k* = 2) in Domain 3 (i.e., bias due to missing outcome data). Studies with some concerns or high concerns in the overall risk of bias were due to Domain 3 (e.g., large amount of missing data), Domain 4 (e.g., self‐assessment of physical activity), or Domain 5 (e.g., lack of pre‐specified plan for measurement and analysis).

### 3.4 Publication bias

**FIGURE 3:**
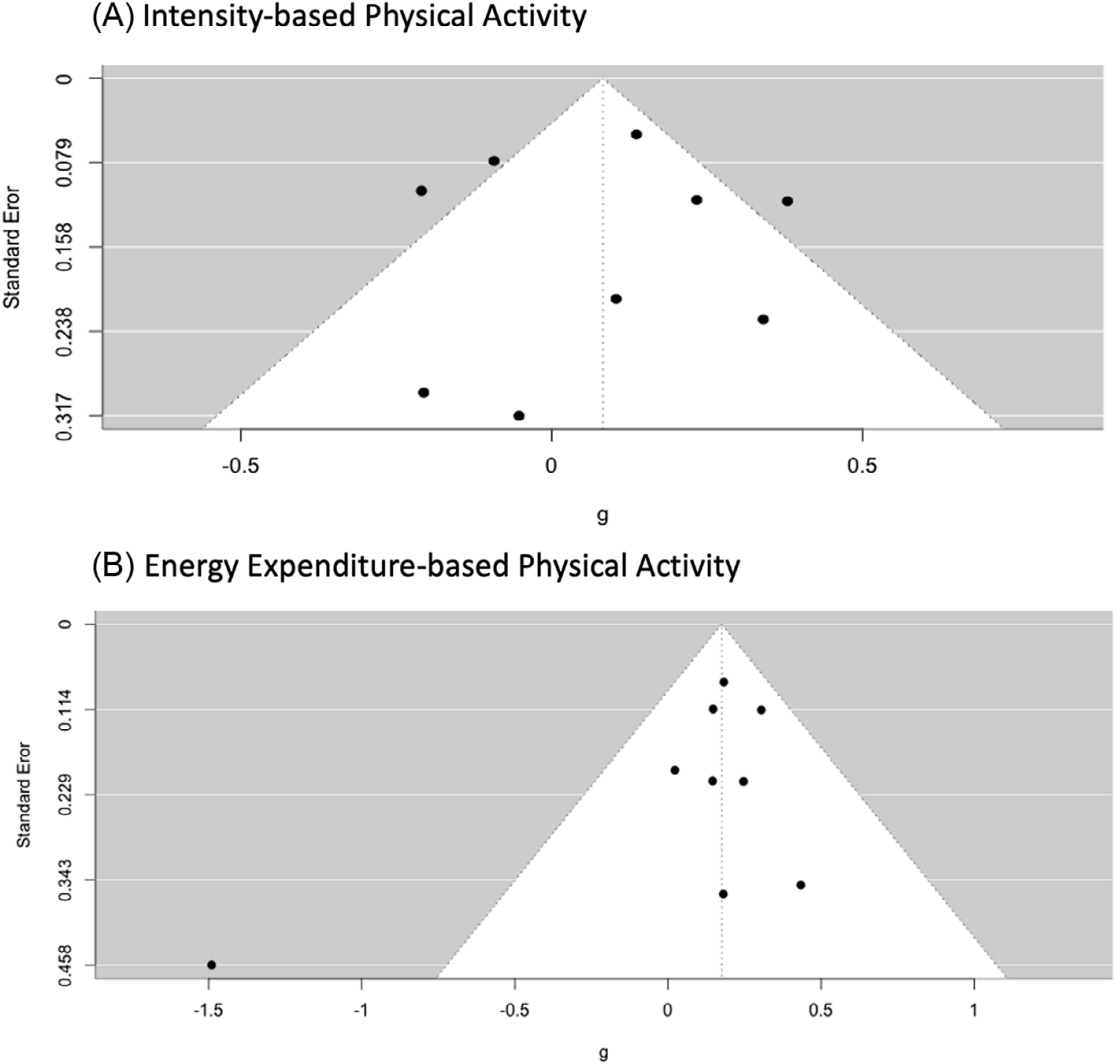
Funnel plot of individual study effect estimates for the physical activity outcomes.

The funnel plots are provided in this manuscript to illustrate the relationship between effect sizes and their precisions, separately by intensity‐based physical activity (see Figure 3A) and energy expenditure‐based physical activity (see Figure 3B). Upon examination of the funnel plots for asymmetry, fit appeared that there might be no presence of publication bias for each physical activity outcome. Furthermore, multiple tests for publication bias provided supporting evidence for the same conclusion regarding each physical activity outcome. These tests included Egger's test of intercept (*p* = 0.88 for intensity‐based physical activity, *p* = 0.09 for energy expenditure‐based physical activity) and the Kendall's tau values (*p* = 0.92 for intensity‐based physical activity, *p* = 1.00 for energy expenditure‐based physical activity).

### 3.5 Intensity‐based physical activity

#### 3.5.1 Overall analysis

**FIGURE 4:**
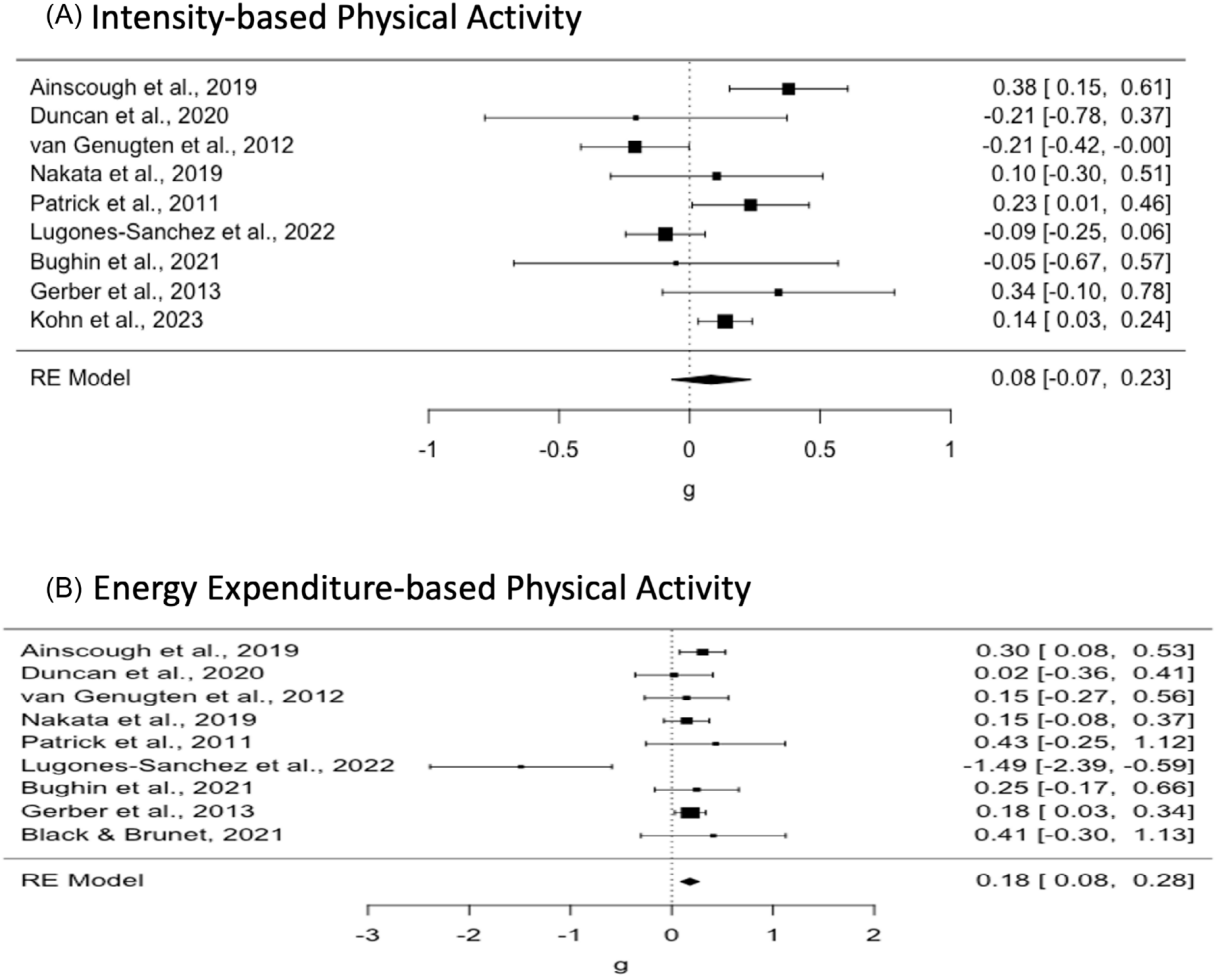
Forest plot of study effects for the physical activity outcomes.

Considering the significant between‐study variability (*Q* (8) = 24.56, *p* < 0.01, I^2^ = 70.68, Birge Ratio = 3.41), we estimated the overall effect using the random‐effects model. The overall effect was calculated as 0.08 (*SE* = 0.08, *z* = 1.06, *p* = 0.29, 95% CI: −0.07 and 0.23). This effect was not statistically significant. The difference in gain scores between the experimental and control groups was found to be small. The experimental group showed a slightly higher gain of 0.08 of a standard deviation, compared to the control group. This suggested a small, albeit nonsignificant, difference at the posttest between the experimental and control groups. Forest plot of study effects for intensity‐based physical activity is provided in Figure 4A.

#### 3.5.2 Moderator analysis

A series of moderator analyses using the mixed‐effects model was conducted to explore whether the intervention effect on intensity‐based physical activity varied based on different moderators. The moderators used in the analysis were (a) sampling method, (b) assignment method, (c) whether matching was used or not, (d) mean age, (e) risk of bias, (f) percentage of female, (g) ethnicity, (h) weight status at baseline, (i) intervention duration, (j) theory use, and (k) the presence of a weight loss and/or management purpose. Among these moderators, the intervention effects on intensity‐based physical activity showed significant difference depending on ethnicity (*Q* (2) = 6.96, *p* = 0.05). Specifically, significant intervention effects were observed only in studies that included participants with mixed ethnic backgrounds (*k*_*effect*_ = 2, $\bar{g}$ = 0.30, *SE* = 0.08, *p* < 0.001, 95% CI: 0.14 and 0.47).

### 3.6 Energy expenditure‐based physical activity

#### 3.6.1 Overall analysis

Considering the significant between‐study variability (*Q* (8) = 15.84, *p* < 0.05, I^2^ = 49.51%, Birge Ratio = 1.98), we estimated the overall effect under the random‐effects model. The overall effect was calculated as 0.18 (*SE* = 0.05, *z* = 3.55, *p* < 0.001, 95% CI: 0.08 and 0.28). This effect was statistically significant. The difference in gain scores between the experimental and control groups was found to be small to moderate. The experimental group showed higher gain of 0.18 of a standard deviation, compared to the control group. This suggested a small to moderate difference at the posttest between the experimental and control groups. Forest plot of study effects for energy expenditure‐based physical activity is provided in Figure 4B.

#### 3.6.2 Moderator analysis

A series of moderator analyses using the mixed‐effects model was conducted to explore whether the intervention effect on energy expenditure‐based physical activity varied based on different moderators. The moderators used in the analysis were (a) sampling method, (b) assignment method, (c) whether matching was used or not, (d) mean age, (e) risk of bias, (f) percentage of female, (g) ethnicity, (h) weight status at baseline, (i) intervention duration, (j) theory use, and (k) the presence of a weight loss and/or management purpose. Among these moderators, the intervention effects on energy expenditure‐based physical activity showed significant difference depending on weight status at baseline (*Q* (3) = 14.20, *p* < 0.001). Specifically, significant intervention effects were observed only in studies that included both adults with overweight and adults with obesity (*k*_*effect*_ = 7, $\bar{g}$ = 0.20, *SE* = 0.05, *p* < 0.01, 95% CI: 0.11 and 0.31), but not in studies that only included either adults with overweight or adults with obesity.

## 4 DISCUSSION

The purpose of this study was to calculate the effects of recent eHealth interventions to promote physical activity in adults with obesity. The effects of recent eHealth interventions depended on the type of outcome variable: intensity‐based physical activity or energy expenditure‐based physical activity. The overall effects of recent eHealth interventions on the physical activity outcomes in adults with obesity were positive and ranged from small to medium in size. Ethnicity and weight status at baseline moderated the effects of recent eHealth interventions on the physical activity outcomes. Results from this meta‐analysis provided some evidence for both the utility of, and possible improvements to, eHealth interventions to promote health‐enhancing physical activity in adults with obesity.

This meta‐analysis showed the difference in the physical activity outcomes, in which recent eHealth interventions had a statistically significant positive effect on energy expenditure‐based physical activity but not intensity‐based physical activity. One explanation for the differing results could be the distinct nature of the outcome variables. The two types of physical activity outcomes may reflect different aspects of physical activity behavior. Intensity‐based physical activity requires a certain threshold of moderate‐ or vigorous‐intensity activity to be recorded.^72^, ^73^ This may possibly make intensity‐based physical activity not sensitive to subtle changes by the eHealth interventions. On the other hand, energy expenditure‐based physical activity is an outcome that considers the energy expenditure associated with various activities,^57^ not requiring different physical activity intensity categories. Energy expenditure‐based physical activity may be sensitive to detecting any changes in physical activity by the eHealth interventions. Another explanation for the differing results could be eHealth intervention emphasis. The eHealth interventions may have been designed with a specific focus on increasing overall energy expenditure or encouraging a broader range of physical activities (e.g., work‐, transport‐, domestic‐, and leisure‐related physical activity) rather than solely targeting different physical activity intensity categories (e.g., moderate‐ or vigorous‐intensity activity). For example, the interventions could have promoted lifestyle changes that promote daily energy expenditure, such as walking, taking the stairs, or reducing sedentary behavior. This may lead to the difference in the meta‐analytic results. Thus, we suspect that the variations in results observed in this meta‐analysis may be attributed to the distinct nature of the outcome variables and/or the eHealth intervention emphasis.

The positive and significant effects of recent eHealth interventions found in this meta‐analysis substantiate the conclusions of the 2018 physical activity guidelines advisory committee scientific report regarding the effects of eHealth interventions on physical activity in adult populations.^8^ The physical activity guidelines advisory committee concluded that eHealth interventions had a small but consistently positive effect in increasing physical activity levels in the unspecified adult population based on previous relevant meta‐analyses.^18^, ^21^ It was also found that eHealth intervention effects were larger in studies that initially screened participants and enrolled only those classified as insufficiently active or sedentary, compared to studies that did not screen participants for physical activity level.^21^ Consistent with the previous studies, this meta‐analysis demonstrates that eHealth interventions have at least a small but positive effect on promoting physical activity specifically in adults with obesity. The study finding holds significance because adults with obesity can greatly benefit from promoting their physical activity, leading to various benefits (e.g., enhanced blood pressure, body composition improvements), even in the absence of weight loss.^8^, ^9^

Regarding the moderation effects on intensity‐based physical activity, significant effects of the interventions were observed in studies with: mixed ethnic backgrounds. One explanation for the moderation effect of mixed ethnic backgrounds could be that there may be more variability (i.e., less restriction in range on the outcome) in the effects of eHealth intervention on physical activity within heterogeneous ethnic groups, compared to homogeneous ethnic groups. Participants from diverse ethnic backgrounds may have different levels of physical activity, on average, influenced by a range of cultural influences, practices, and beliefs. Thus, mixed ethnic backgrounds may create a more diverse outcome variable, allowing eHealth interventions to address a broader spectrum of factors that influence physical activity. In contrast, more homogeneous ethnic backgrounds may exhibit more uniform cultural characteristics, potentially limiting the effects of eHealth interventions that are not designed with specific cultural adaptations. Interventions for homogeneous ethnic backgrounds can, however, promote behavioral change if cultural adaptations are implemented in the intervention.^74^

Regarding the moderation effects on energy expenditure‐based physical activity, significant effects of the interventions were observed solely in studies encompassing both adults with overweight and obesity. Adults with excess weight, whether categorized as overweight or obesity, encounter elevated risks of diverse health issues such as cancer, cardiovascular diseases, type 2 diabetes mellitus, and hypertension.^46^ Therefore, incorporating eHealth physical activity interventions that encompass adults with overweight can serve as a preventive strategy to mitigate their health vulnerabilities. Previous reviews on physical activity interventions for adults with obesity noted the involvement of adults with overweight,^20^, ^47^, ^48^ yet no rationale or clarification was provided for this inclusion. The moderation finding in this meta‐analysis could serve as an explicit justification for including adults with overweight into online physical activity interventions designed for adults with obesity. We believe that the moderation effects found in this meta‐analysis (i.e., ethnicity, weight status) can be used to improve the utility of eHealth interventions to promote physical activity in adults with obesity.

Although theory use in the eHealth interventions did not moderate the effects on the physical activity outcomes in this meta‐analysis, the theoretical approach can provide a unifying framework from which behavior changes can be well understood and further promoted.^75^, ^76^ With theory use in eHealth interventions (e.g., developing interventions based on self‐efficacy theory), researchers may both measure and evaluate a theoretical modifiable variable (e.g., physical activity self‐efficacy) that is expected to lead to the change in physical activity. In this meta‐analysis, only half of the studies explicitly reported the use of behavioral theory in describing their eHealth interventions. This may indicate that theory use is not prevalent in studies using eHealth interventions to promote physical activity in adults with obesity. This could result in a lack of clear understanding regarding the ways in which eHealth interventions promote physical activity in adults with obesity. Theoretical perspectives may offer a cohesive framework that enhances the comprehension and facilitation of physical activity changes in eHealth interventions for adults with obesity.

The evaluation of risk of bias showed that most of the included studies may have at least some concerns in the overall risk of bias. To be specific, the majority of studies had some concerns or high concerns in the overall risk of bias due to three domains: Domain 3 (i.e., bias due to missing outcome data), Domain 4 (i.e., bias in measurement of the outcome), and Domain 5 (i.e., bias in selection of the reported result). The risk of bias in this meta‐analysis is similar with previous meta‐analyses of behavioral interventions in which at least 70% of studies seemed to have high concerns in overall risk of bias due to the aforementioned domains.^18^, ^77^ It is important to reduce bias in the eHealth intervention studies in the future to better assess the impact of the interventions on the physical activity of adults with obesity.

Using various strategies can reduce the risk of bias in future eHealth intervention studies. For the bias from missing data and selection of the reported result, researchers are recommended to precisely report missing data and a pre‐specified plan for outcome measurements and analyses. The use of the Consolidated Standards of Reporting Trials statement has the potential to improve reporting and provides a basis for evaluating the validity and applicability of behavioral interventions.^78^, ^79^ Additionally, the bias due to missing outcome data can be reduced by using modern analytic methods for missing data.^80^ For the bias in measurement of the outcome, using device‐based physical activity assessments is recommended in future eHealth intervention studies. This is because adults with obesity may have a response bias due to social desirability.^81^, ^82^ However, the majority of the studies in this meta‐analysis used questionnaire‐based assessment (i.e., self‐reports). Increased use of device‐based assessment may help ameliorate some of the challenges that questionnaire‐based assessment has and reduce self‐report‐based bias. Employing different approaches can mitigate bias in forthcoming studies on eHealth interventions to promote physical activity in adults with obesity.

We provided some recommendations for future eHealth intervention studies. First, future eHealth interventions can integrate behavioral change techniques (e.g., self‐monitoring and goal setting) that have been documented as effective in promoting physical activity in adults with obesity.^31^, ^32^, ^33^ Nevertheless, it remained uncertain whether the recent eHealth interventions employed the effective behavioral change techniques for adults with obesity. It is also recommended to explicitly report any behavioral change techniques used in the interventions in the published manuscript, for example, by using hierarchically structured taxonomy of techniques.^83^ Second, a theory application approach in developing and testing eHealth physical activity interventions can be more frequently and carefully adopted in the future. If a theoretical conceptual model is employed, future research could assess and analyze the pertinent theoretical constructs through mediation (or indirect effect) analysis. This may help understand the mechanisms behind the intervention effects. Third, the Community Preventive Services Task Force recommends implementing physical activity interventions within a more broadly focused weight management program.^84^ The practical aspects of eHealth interventions, such as their easy accessibility and affordability, contribute to the feasibility of the intervention use by patients in a healthcare center. If implemented, adults with obesity could enhance their health through both eHealth physical activity interventions and weight management programs, with access to additional healthcare services like medication and dietary support.

We are aware of at least three limitations of this meta‐analysis. First, the evaluation of risk of bias in this meta‐analysis was possibly subjective. The limitation was partially addressed by carefully following the Rob 2 guideline by the two independent raters.^55^ Second, some researchers may use a different age range for adult populations (e.g., 18–70 years) instead of 18–65 years. The age break particularly between adults and older adults is not well‐established but may generally center on retirement.^8^ Third, the recent eHealth intervention studies had the variability in the measurement units and assessment methods used for quantifying physical activity (e.g., MVPA, MET, questionnaire‐based assessment, device‐based assessment). The variability may create the potential lack of consistency and comparability regarding the effects of recent eHealth interventions on physical activity in adults with obesity. This limitation may highlight the need for establishing recommended approaches to measuring and reporting physical activity in adults with obesity to enhance the reliability and validity of meta‐analytic results.

## 5 CONCLUSION

We believe that this meta‐analysis makes a meaningful contribution to understanding the effects of recent eHealth interventions on physical activity in adults with obesity. In addition to providing coherent support for the effects of eHealth interventions on physical activity in general adult populations,^8^, ^18^, ^21^ our analyses suggest that eHealth interventions have at least a small but positive effect on promoting health‐enhancing physical activity in adults with obesity. Also, the moderation effects (e.g., weight status at baseline) found in this study can be used to improve the utility of eHealth interventions to promote physical activity in adults with obesity. Physical activity promotion can be an effective means for adults with obesity to enhance their health, irrespective of whether weight loss is achieved or not. This meta‐analysis may provide contemporary and important research findings to help researchers and practitioners who develop, implement, and evaluate eHealth interventions to promote physical activity in adults with obesity.

## CONFLICT OF INTEREST STATEMENT

Authors have no conflict of interest to declare.

## Supporting information


**Table S1.** Database, Search Keywords, and Search Results.
**Table S2**. Reasons for Exclusion of Each Study During Second‐Step Full‐Text Screening of Retrieved Articles.
**Table S3**. Characteristic Summary of Included Studies.
