# Supplementary material for: A meta‐analysis of eHealth interventions to promote physical activity in young, middle‐aged, and late middle‐aged adults with obesity or overweight
Source: Obes Rev. 2025 Jan 10;26(6):e13898. doi: 10.1111/obr.13898 (PMC12069164; doi:10.1111/obr.13898)
Supplement: Supplementary file 1 — Table S1. Database, Search Keywords, and Search Results. Table S2. Reasons for Exclusion of Each Study During Second‐Step Full‐Text Screening of Retrieved Articles. Table S3. Characteristic Summary of Included Studies. [file OBR-26-e13898-s001.pdf]

# A Meta-analysis of eHealth Interventions to Promote Physical Activity in Young, Middle-Aged, and Late Middle-Aged Adults with Obesity or Overweight

Seungmin Lee, Department of Kinesiology, Iowa State University, seunglee@iastate.edu, https://orcid.org/0000-0003-3463-4004; Soyeon Ahn, Department of Educational and Psychological Studies, University of Miami, s.ahn@miami.edu, https://orcid.org/0000-0003-2581-306X; Priya Patel, Department of Kinesiology, California State University Fullerton, priya.patel@fullerton.edu, https://orcid.org/0000-0003-4382-0300; Nicholas D. Myers, Department of Kinesiology, Michigan State University, myersni1@msu.edu, https://orcid.org/0000-0001-9069-9940

Correspondence concerning this article should be addressed to Seungmin Lee, Department of Kinesiology, Iowa State University, 103G Forker Building, 534 Wallace Road, Ames, IA 50011 E-mail: seunglee@iastate.edu

Table 1s

*Database, Search Keywords, and Search Results*

| database | search keywords | search results |
|---|---|---|
| CINAHL | S1: physical activity OR physical training OR exercis* OR aerobic OR walk OR running OR bicycle OR cycling OR swim OR yoga OR danc* OR gardening<br>S2: eHealth OR mHelath OR digital OR computer OR web OR online OR internet OR mobile application OR phone OR email OR exergame* OR acceleromet*<br>S3: adults with obesity OR adults with overweight OR obese adult OR obese patient<br>S4: S1 AND S2 AND S3 | 255 results (01/01/2010 - 05/23/2022)<br>+<br>36 results (05/24/2022 - 10/29/2024) |
| Cochrane | #1: (physical activity):ti,ab,kw OR (physical training):ti,ab,kw OR (exercis*):ti,ab,kw OR (aerobic):ti,ab,kw AND (walk):ti,ab,kw<br>#2: (digital):ti,ab,kw OR (online):ti,ab,kw OR (internet):ti,ab,kw OR (web):ti,ab,kw OR (app):ti,ab,kw<br>#3: (adults with obesity):ti,ab,kw OR (adults with overweight):ti,ab,kw OR (obese adult):ti,ab,kw OR (obese patient):ti,ab,kw<br>#4: #1 AND #2 AND #3 | 946 results (01/01/2010 - 05/23/2022)<br>+<br>136 results (05/24/2022 - 10/29/2024) |
| Embase | #1: ('physical activity':ti,ab,kw OR 'physical training':ti,ab,kw OR exercis*:ti,ab,kw OR aerobic:ti,ab,kw OR walk:ti,ab,kw OR running:ti,ab,kw OR bicycle:ti,ab,kw OR cycling:ti,ab,kw OR swim:ti,ab,kw OR yoga:ti,ab,kw OR danc*:ti,ab,kw OR gardening:ti,ab,kw) AND ([controlled clinical trial]/lim OR [randomized controlled trial]/lim)<br>#2: (ehealth:ti,ab,kw OR mhelath:ti,ab,kw OR digital:ti,ab,kw OR computer:ti,ab,kw OR web:ti,ab,kw OR online:ti,ab,kw OR internet:ti,ab,kw OR 'mobile application':ti,ab,kw OR phone:ti,ab,kw OR 'text messag*':ti,ab,kw OR email:ti,ab,kw OR 'electronic mail':ti,ab,kw OR multimedia:ti,ab,kw OR exergame*:ti,ab,kw OR acceleromet*:ti,ab,kw OR pedometer:ti,ab,kw) AND ([controlled clinical trial]/lim OR [randomized controlled trial]/lim)<br>#3: ('adults with obesity' OR (('adults'/exp OR adults) AND with AND ('obesity'/exp OR obesity)) OR 'adults with overweight' OR (('adults'/exp OR adults) AND with AND ('overweight'/exp OR overweight)) OR 'obese adult' OR (obese AND ('adult'/exp OR adult)) OR 'obese patient'/exp OR 'obese patient' OR (obese AND ('patient'/exp OR patient))) AND ([controlled clinical trial]/lim OR [randomized controlled trial]/lim)<br>#4: #1 AND #2 AND #3 | 945 results (01/01/2010 - 05/23/2022)<br>+<br>148 results (05/24/2022 - 10/29/2024) |

| database | search keywords | search results |
|---|---|---|
| PsycINFO | S1: (noft(physical activity) OR noft(physical training) OR noft(exercis*) OR noft(aerobic) OR noft(walk) OR noft(running) OR noft(bicycle) OR noft(cycling) OR noft(swim) OR noft(yoga)) AND PEER(yes)<br>S2: (noft(eHealth) OR noft(mHelath) OR noft(digital) OR noft(computer) OR noft(web) OR noft(online) OR noft(internet) OR noft(app) OR noft(phone) OR noft(email)) AND PEER(yes)<br>S3: ((adults with obesity) OR (adults with overweight) OR (obese adult) OR (obese patient)) AND PEER(yes)<br>S4: (S1 AND S2 AND S3) AND me.exact("Empirical Study" NOT ("Meta Analysis" OR "Literature Review" OR "Systematic Review" OR "Brain Imaging" OR "Mathematical Model")) | 192 results (01/01/2010 - 05/23/2022)<br>+<br>137 results (05/24/2022 - 10/29/2024) |
| PubMed | #1: ((((((((((physical activity) OR (physical training)) OR (exercis*)) OR (aerobic)) OR (walk)) OR (running)) OR (bicycle)) OR (cycling)) OR (swim)) OR (yoga)) OR (danc*)) OR (gardening)<br>#2: ((((((((((((((eHealth) OR (mHelath)) OR (digital)) OR (computer)) OR (web)) OR (online)) OR (internet)) OR (mobile application)) OR (phone)) OR (text messag*)) OR (email)) OR (electronic mail)) OR (multimedia)) OR (exergame*)) OR (acceleromet*)) OR (pedometer)<br>#3: (((adults with obesity) OR (adults with overweight)) OR (obese adult)) OR (obese patient)<br>#4: #1 AND #2 AND #3 | 1554 results (01/01/2010 - 05/23/2022)<br>+<br>534 results (05/24/2022 - 10/29/2024) |

Table 2s

*Reasons for Exclusion of Each Study During Second-Step Full-Text Screening of Retrieved Articles*

| reference | 1. year/ articles | 2. eHealth for PA | 3. RCT to test eHealth | 4. age 18-65 | 5. BMI 25 or higher | 6. PA assessment | 7. sufficient statistical information | 8. not pilot or feasibility studies | 9. not gray literature | eligibility |
|---|---|---|---|---|---|---|---|---|---|---|
| 1 | 1 | 0 | 1 | 1 | 1 | 1 | 0 | 1 | 1 | 0 |
| 2 | 0 | 0 | 1 | 1 | 0 | 1 | 0 | 1 | 1 | 0 |
| 3 | 1 | 0 | 0 | 1 | 1 | 1 | 0 | 1 | 1 | 0 |
| 4 | 1 | 0 | 1 | 1 | 1 | 1 | 1 | 1 | 1 | 0 |
| 5 | 1 | 1 | 1 | 1 | 1 | 1 | 1 | 1 | 1 | 1 |
| 6 | 1 | 0 | 1 | 0 | 1 | 1 | 1 | 1 | 1 | 0 |
| 7 | 1 | 1 | 1 | 1 | 1 | 1 | 1 | 0 | 1 | 0 |
| 8 | 1 | 0 | 1 | 0 | 1 | 1 | 1 | 0 | 1 | 0 |
| 9 | 1 | 0 | 1 | 0 | 1 | 1 | 1 | 1 | 1 | 0 |
| 10 | 1 | 0 | 1 | 1 | 1 | 1 | 1 | 1 | 1 | 0 |
| 11 | 1 | 1 | 1 | 0 | 1 | 1 | 0 | 1 | 1 | 0 |
| 12 | 1 | 1 | 1 | 1 | 1 | 0 | 0 | 1 | 1 | 0 |
| 13 | 0 | 1 | 1 | 1 | 1 | 0 | 0 | 1 | 1 | 0 |
| 14 | 1 | 1 | 1 | 0 | 1 | 1 | 1 | 1 | 1 | 0 |
| 15 | 1 | 1 | 0 | 0 | 1 | 1 | 1 | 1 | 1 | 0 |
| 16 | 1 | 0 | 1 | 1 | 1 | 1 | 0 | 1 | 1 | 0 |
| 17 | 1 | 1 | 1 | 0 | 1 | 0 | 0 | 1 | 1 | 0 |
| 18 | 1 | 0 | 1 | 0 | 1 | 1 | 0 | 1 | 1 | 0 |
| 19 | 1 | 0 | 1 | 0 | 1 | 1 | 0 | 1 | 1 | 0 |
| 20 | 1 | 0 | 1 | 1 | 1 | 1 | 1 | 0 | 1 | 0 |
| 21 | 1 | 0 | 1 | 0 | 1 | 1 | 1 | 0 | 1 | 0 |
| 22 | 1 | 1 | 1 | 0 | 1 | 1 | 1 | 0 | 1 | 0 |
| 23 | 1 | 1 | 1 | 0 | 1 | 1 | 1 | 1 | 1 | 0 |

| reference | 1. year/ articles | 2. eHealth for PA | 3. RCT to test eHealth | 4. age 18-65 | 5. BMI 25 or higher | 6. PA assessment | 7. sufficient statistical information | 8. not pilot or feasibility studies | 9. not gray literature | eligibility |
|---|---|---|---|---|---|---|---|---|---|---|
| 24 | 1 | 1 | 1 | 1 | 0 | 1 | 0 | 1 | 1 | 0 |
| 25 | 0 | 1 | 1 | 1 | 0 | 1 | 0 | 1 | 1 | 0 |
| 26 | 0 | 1 | 0 | 0 | 0 | 1 | 0 | 1 | 1 | 0 |
| 27 | 1 | 1 | 1 | 0 | 1 | 1 | 1 | 1 | 1 | 0 |
| 28 | 1 | 0 | 0 | 0 | 1 | 1 | 0 | 0 | 1 | 0 |
| 29 | 1 | 0 | 1 | 1 | 1 | 1 | 1 | 0 | 1 | 0 |
| 30 | 1 | 0 | 1 | 1 | 1 | 1 | 0 | 1 | 1 | 0 |
| 31 | 1 | 1 | 1 | 0 | 1 | 1 | 0 | 1 | 1 | 0 |
| 32 | 0 | 1 | 1 | 1 | 1 | 1 | 1 | 1 | 1 | 0 |
| 33 | 1 | 0 | 0 | 0 | 0 | 1 | 1 | 0 | 1 | 0 |
| 34 | 1 | 1 | 1 | 0 | 1 | 1 | 0 | 0 | 1 | 0 |
| 35 | 1 | 0 | 1 | 1 | 1 | 1 | 1 | 1 | 1 | 0 |
| 36 | 0 | 0 | 1 | 0 | 1 | 1 | 1 | 1 | 1 | 0 |
| 37 | 1 | 1 | 1 | 1 | 1 | 1 | 1 | 1 | 1 | 1 |
| 38 | 1 | 0 | 1 | 0 | 1 | 1 | 1 | 1 | 1 | 0 |
| 39 | 1 | 0 | 1 | 0 | 1 | 1 | 0 | 1 | 1 | 0 |
| 40 | 1 | 1 | 1 | 0 | 1 | 1 | 1 | 1 | 1 | 0 |
| 41 | 1 | 1 | 0 | 0 | 1 | 1 | 1 | 1 | 1 | 0 |
| 42 | 1 | 1 | 1 | 1 | 1 | 1 | 1 | 0 | 1 | 0 |
| 43 | 1 | 1 | 1 | 1 | 1 | 1 | 0 | 1 | 1 | 2 |
| 44 | 1 | 0 | 1 | 0 | 1 | 1 | 0 | 0 | 1 | 0 |
| 45 | 1 | 1 | 1 | 0 | 1 | 1 | 1 | 1 | 1 | 0 |
| 46 | 1 | 0 | 1 | 1 | 1 | 1 | 1 | 1 | 1 | 0 |
| 47 | 1 | 1 | 1 | 0 | 1 | 1 | 0 | 1 | 1 | 0 |
| 48 | 1 | 1 | 1 | 0 | 1 | 1 | 1 | 0 | 1 | 0 |
| 49 | 0 | 1 | 1 | 0 | 1 | 1 | 0 | 1 | 1 | 0 |

| reference | 1. year/ articles | 2. eHealth for PA | 3. RCT to test eHealth | 4. age 18-65 | 5. BMI 25 or higher | 6. PA assessment | 7. sufficient statistical information | 8. not pilot or feasibility studies | 9. not gray literature | eligibility |
|---|---|---|---|---|---|---|---|---|---|---|
| 50 | 0 | 0 | 1 | 0 | 0 | 1 | 1 | 1 | 1 | 0 |
| 51 | 1 | 1 | 1 | 0 | 1 | 1 | 0 | 1 | 1 | 0 |
| 52 | 1 | 1 | 1 | 0 | 1 | 1 | 0 | 1 | 1 | 0 |
| 53 | 1 | 1 | 1 | 1 | 1 | 1 | 0 | 1 | 1 | 0 |
| 54 | 1 | 1 | 1 | 0 | 1 | 0 | 0 | 1 | 1 | 0 |
| 55 | 1 | 0 | 1 | 1 | 0 | 1 | 0 | 0 | 1 | 0 |
| 56 | 1 | 0 | 1 | 1 | 1 | 0 | 0 | 1 | 1 | 0 |
| 57 | 1 | 1 | 1 | 1 | 1 | 0 | 0 | 1 | 1 | 0 |
| 58 | 1 | 1 | 1 | 0 | 1 | 1 | 1 | 1 | 1 | 0 |
| 59 | 1 | 1 | 0 | 1 | 1 | 1 | 0 | 1 | 1 | 0 |
| 60 | 0 | 0 | 1 | 1 | 1 | 1 | 1 | 1 | 1 | 0 |
| 61 | 1 | 0 | 1 | 1 | 1 | 1 | 1 | 1 | 1 | 0 |
| 62 | 1 | 0 | 1 | 0 | 1 | 1 | 1 | 1 | 1 | 0 |
| 63 | 0 | 1 | 1 | 0 | 1 | 1 | 0 | 1 | 1 | 0 |
| 64 | 1 | 1 | 0 | 1 | 1 | 1 | 1 | 0 | 1 | 0 |
| 65 | 1 | 0 | 1 | 1 | 1 | 1 | 0 | 1 | 1 | 0 |
| 66 | 1 | 0 | 1 | 0 | 0 | 1 | 0 | 1 | 1 | 0 |
| 67 | 1 | 0 | 1 | 1 | 1 | 1 | 0 | 0 | 1 | 0 |
| 68 | 1 | 1 | 0 | 1 | 1 | 1 | 0 | 0 | 1 | 0 |
| 69 | 1 | 1 | 1 | 1 | 1 | 1 | 1 | 1 | 1 | 1 |
| 70 | 1 | 1 | 1 | 0 | 1 | 1 | 0 | 1 | 1 | 0 |
| 71 | 1 | 0 | 0 | 0 | 1 | 1 | 0 | 1 | 1 | 0 |
| 72 | 1 | 1 | 1 | 1 | 1 | 1 | 1 | 1 | 1 | 1 |
| 73 | 0 | 0 | 1 | 0 | 1 | 1 | 0 | 1 | 1 | 0 |
| 74 | 0 | 1 | 1 | 1 | 1 | 1 | 1 | 1 | 1 | 0 |
| 75 | 1 | 0 | 0 | 0 | 0 | 1 | 1 | 1 | 1 | 0 |

| reference | 1. year/ articles | 2. eHealth for PA | 3. RCT to test eHealth | 4. age 18-65 | 5. BMI 25 or higher | 6. PA assessment | 7. sufficient statistical information | 8. not pilot or feasibility studies | 9. not gray literature | eligibility |
|---|---|---|---|---|---|---|---|---|---|---|
| 76 | 1 | 1 | 1 | 1 | 1 | 0 | 0 | 1 | 1 | 0 |
| 77 | 0 | 1 | 1 | 1 | 1 | 1 | 0 | 1 | 1 | 0 |
| 78 | 0 | 0 | 1 | 1 | 1 | 1 | 0 | 1 | 1 | 0 |
| 79 | 1 | 1 | 1 | 1 | 1 | 1 | 0 | 1 | 1 | 2 |
| 80 | 1 | 1 | 1 | 0 | 1 | 0 | 0 | 0 | 1 | 0 |
| 81 | 1 | 1 | 1 | 0 | 1 | 1 | 0 | 1 | 1 | 0 |
| 82 | 1 | 0 | 1 | 1 | 1 | 1 | 0 | 1 | 1 | 0 |
| 83 | 0 | 1 | 1 | 1 | 1 | 1 | 0 | 1 | 1 | 0 |
| 84 | 1 | 1 | 1 | 0 | 1 | 1 | 0 | 1 | 1 | 0 |
| 85 | 1 | 1 | 1 | 1 | 1 | 1 | 1 | 1 | 1 | 1 |
| 86 | 1 | 1 | 1 | 1 | 1 | 1 | 1 | 1 | 1 | 1 |
| 87 | 1 | 1 | 0 | 1 | 1 | 1 | 1 | 1 | 1 | 0 |
| 88 | 0 | 0 | 1 | 0 | 0 | 1 | 0 | 1 | 0 | 0 |
| 89 | 1 | 1 | 0 | 0 | 0 | 0 | 0 | 1 | 1 | 0 |
| 90 | 1 | 1 | 1 | 0 | 1 | 1 | 1 | 1 | 1 | 0 |
| 91 | 1 | 1 | 1 | 1 | 1 | 1 | 1 | 1 | 1 | 1 |
| 92 | 1 | 1 | 1 | 1 | 1 | 1 | 1 | 1 | 1 | 1 |
| 93 | 1 | 0 | 1 | 1 | 1 | 1 | 1 | 0 | 1 | 0 |
| 94 | 0 | 1 | 0 | 1 | 1 | 1 | 1 | 1 | 1 | 0 |
| 95 | 1 | 0 | 1 | 0 | 0 | 1 | 1 | 1 | 1 | 0 |
| 96 | 0 | 1 | 1 | 0 | 1 | 1 | 1 | 0 | 1 | 0 |
| 97 | 0 | 1 | 0 | 0 | 1 | 1 | 0 | 0 | 1 | 0 |
| 98 | 0 | 0 | 1 | 0 | 1 | 1 | 1 | 1 | 1 | 0 |
| 99 | 1 | 0 | 1 | 0 | 1 | 1 | 1 | 1 | 1 | 0 |
| 100 | 1 | 1 | 0 | 1 | 1 | 1 | 0 | 1 | 1 | 0 |
| 101 | 1 | 1 | 1 | 1 | 1 | 1 | 1 | 1 | 1 | 1 |

| reference | 1. year/ articles | 2. eHealth for PA | 3. RCT to test eHealth | 4. age 18-65 | 5. BMI 25 or higher | 6. PA assessment | 7. sufficient statistical information | 8. not pilot or feasibility studies | 9. not gray literature | eligibility |
|---|---|---|---|---|---|---|---|---|---|---|
| 102 | 1 | 1 | 1 | 1 | 1 | 1 | 1 | 0 | 1 | 0 |
| 103 | 1 | 1 | 1 | 1 | 1 | 1 | 0 | 1 | 1 | 2 |
| 104 | 1 | 1 | 0 | 0 | 1 | 1 | 1 | 0 | 1 | 0 |
| 105 | 1 | 0 | 1 | 0 | 0 | 1 | 1 | 0 | 1 | 0 |
| 106 | 1 | 0 | 1 | 0 | 1 | 1 | 1 | 0 | 1 | 0 |
| 107 | 0 | 1 | 1 | 0 | 1 | 1 | 1 | 1 | 1 | 0 |
| 108 | 1 | 1 | 1 | 0 | 1 | 1 | 1 | 1 | 1 | 0 |
| 109 | 1 | 0 | 0 | 1 | 1 | 1 | 1 | 0 | 1 | 0 |
| 110 | 1 | 0 | 1 | 1 | 1 | 1 | 0 | 1 | 1 | 0 |
| 111 | 1 | 1 | 1 | 1 | 1 | 1 | 1 | 1 | 1 | 1 |
| 112 | 1 | 0 | 1 | 1 | 1 | 1 | 1 | 1 | 1 | 0 |
| 113 | 1 | 0 | 1 | 0 | 0 | 1 | 1 | 0 | 1 | 0 |
| 114 | 1 | 0 | 0 | 0 | 1 | 1 | 1 | 1 | 1 | 0 |
| 115 | 1 | 0 | 1 | 1 | 1 | 1 | 0 | 1 | 1 | 0 |
| 116 | 1 | 1 | 1 | 0 | 1 | 0 | 0 | 1 | 1 | 0 |
| 117 | 1 | 1 | 1 | 0 | 1 | 1 | 1 | 1 | 1 | 0 |
| 118 | 1 | 1 | 1 | 0 | 0 | 1 | 0 | 1 | 1 | 0 |
| 119 | 1 | 1 | 0 | 0 | 1 | 1 | 0 | 1 | 1 | 0 |
| 120 | 1 | 1 | 0 | 1 | 1 | 1 | 0 | 1 | 1 | 0 |
| 121 | 0 | 0 | 1 | 0 | 1 | 1 | 1 | 1 | 1 | 0 |
| 122 | 1 | 0 | 1 | 0 | 1 | 1 | 0 | 1 | 1 | 0 |
| 123 | 1 | 0 | 0 | 1 | 1 | 1 | 1 | 1 | 1 | 0 |
| 124 | 1 | 0 | 0 | 1 | 1 | 1 | 0 | 1 | 1 | 0 |
| 125 | 1 | 1 | 1 | 1 | 1 | 0 | 0 | 1 | 1 | 0 |
| 126 | 0 | 1 | 1 | 0 | 1 | 1 | 0 | 1 | 1 | 0 |
| 127 | 1 | 0 | 0 | 0 | 1 | 1 | 0 | 1 | 1 | 0 |

| reference | 1. year/ articles | 2. eHealth for PA | 3. RCT to test eHealth | 4. age 18-65 | 5. BMI 25 or higher | 6. PA assessment | 7. sufficient statistical information | 8. not pilot or feasibility studies | 9. not gray literature | eligibility |
|---|---|---|---|---|---|---|---|---|---|---|
| 128 | 1 | 0 | 0 | 0 | 0 | 1 | 1 | 1 | 1 | 0 |
| 129 | 1 | 0 | 1 | 0 | 1 | 1 | 1 | 1 | 1 | 0 |
| 130 | 1 | 1 | 1 | 1 | 1 | 1 | 0 | 1 | 1 | 2 |
| 131 | 1 | 1 | 1 | 0 | 1 | 1 | 0 | 1 | 1 | 0 |
| 132 | 1 | 1 | 0 | 0 | 1 | 1 | 1 | 1 | 1 | 0 |
| 133 | 1 | 1 | 1 | 0 | 1 | 1 | 1 | 0 | 1 | 0 |
| 134 | 1 | 0 | 0 | 0 | 0 | 1 | 0 | 1 | 1 | 0 |
| 135 | 1 | 1 | 0 | 0 | 1 | 1 | 0 | 1 | 1 | 0 |
| 136 | 0 | 1 | 1 | 0 | 0 | 1 | 1 | 1 | 1 | 0 |
| 137 | 1 | 1 | 1 | 0 | 1 | 1 | 1 | 1 | 1 | 0 |
| 138 | 1 | 1 | 0 | 0 | 1 | 1 | 1 | 1 | 1 | 0 |
| 139 | 1 | 1 | 0 | 0 | 1 | 1 | 0 | 1 | 1 | 0 |
| 140 | 1 | 1 | 0 | 0 | 1 | 1 | 0 | 1 | 1 | 0 |
| 141 | 1 | 1 | 1 | 1 | 0 | 1 | 1 | 1 | 1 | 0 |
| 142 | 0 | 0 | 1 | 0 | 1 | 1 | 1 | 1 | 1 | 0 |
| 143 | 1 | 1 | 1 | 1 | 1 | 1 | 1 | 0 | 1 | 0 |
| 144 | 1 | 1 | 1 | 0 | 0 | 1 | 1 | 1 | 1 | 0 |
| 145 | 1 | 1 | 1 | 1 | 1 | 0 | 0 | 1 | 1 | 0 |
| 146 | 1 | 1 | 1 | 0 | 0 | 1 | 1 | 1 | 1 | 0 |
| 147 | 1 | 1 | 1 | 1 | 1 | 1 | 1 | 1 | 1 | 1 |
| 148 | 1 | 1 | 1 | 0 | 1 | 1 | 1 | 1 | 1 | 0 |
| 149 | 1 | 1 | 0 | 1 | 1 | 1 | 1 | 1 | 1 | 0 |
| 150 | 1 | 0 | 1 | 1 | 1 | 1 | 1 | 0 | 1 | 0 |
| 151 | 1 | 1 | 0 | 0 | 1 | 1 | 0 | 1 | 1 | 0 |
| 152 | 1 | 1 | 0 | 1 | 0 | 1 | 1 | 1 | 1 | 0 |

| reference | 1. year/ articles | 2. eHealth for PA | 3. RCT to test eHealth | 4. age 18-65 | 5. BMI 25 or higher | 6. PA assessment | 7. sufficient statistical information | 8. not pilot or feasibility studies | 9. not gray literature | eligibility |
|---|---|---|---|---|---|---|---|---|---|---|
| 153 | 1 | 1 | 1 | 0 | 1 | 1 | 0 | 1 | 1 | 0 |
| 154 | 1 | 0 | 1 | 0 | 1 | 0 | 0 | 1 | 1 | 0 |
| 155 | 1 | 1 | 0 | 0 | 1 | 0 | 0 | 1 | 1 | 0 |
| 156 | 1 | 1 | 1 | 1 | 1 | 1 | 0 | 1 | 1 | 2 |
| 157 | 1 | 1 | 1 | 1 | 1 | 1 | 1 | 1 | 1 | 1 |
| 158 | 1 | 1 | 1 | 0 | 1 | 1 | 1 | 1 | 1 | 0 |
| 159 | 1 | 0 | 1 | 0 | 1 | 1 | 0 | 1 | 1 | 0 |
| 160 | 1 | 0 | 1 | 0 | 1 | 1 | 1 | 1 | 1 | 0 |
| 161 | 1 | 1 | 1 | 0 | 1 | 1 | 1 | 1 | 1 | 0 |
| 162 | 1 | 1 | 1 | 1 | 1 | 1 | 0 | 1 | 1 | 2 |
| 163 | 1 | 1 | 0 | 0 | 0 | 1 | 1 | 0 | 1 | 0 |
| 164 | 1 | 1 | 1 | 0 | 1 | 1 | 1 | 0 | 1 | 0 |
| 165 | 1 | 1 | 1 | 0 | 0 | 1 | 1 | 1 | 1 | 0 |
| 166 | 1 | 1 | 0 | 0 | 1 | 1 | 0 | 1 | 1 | 0 |
| 167 | 1 | 0 | 1 | 0 | 1 | 1 | 1 | 1 | 1 | 0 |
| 168 | 1 | 1 | 0 | 1 | 1 | 1 | 0 | 1 | 1 | 0 |
| 169 | 1 | 0 | 0 | 1 | 1 | 1 | 1 | 1 | 1 | 0 |
| 170 | 1 | 1 | 1 | 0 | 1 | 1 | 0 | 1 | 1 | 0 |
| 171 | 1 | 1 | 0 | 0 | 0 | 0 | 0 | 1 | 1 | 0 |
| 172 | 1 | 1 | 0 | 0 | 1 | 1 | 0 | 1 | 1 | 0 |
| 173 | 1 | 1 | 0 | 0 | 0 | 1 | 0 | 1 | 1 | 0 |
| 174 | 1 | 1 | 0 | 0 | 0 | 1 | 0 | 1 | 1 | 0 |
| 175 | 1 | 1 | 1 | 1 | 1 | 0 | 0 | 1 | 1 | 0 |
| 176 | 1 | 1 | 1 | 1 | 0 | 1 | 0 | 1 | 1 | 0 |

| reference | 1. year/ articles | 2. eHealth for PA | 3. RCT to test eHealth | 4. age 18-65 | 5. BMI 25 or higher | 6. PA assessment | 7. sufficient statistical information | 8. not pilot or feasibility studies | 9. not gray literature | eligibility |
|---|---|---|---|---|---|---|---|---|---|---|
| 177 | 1 | 0 | 1 | 0 | 1 | 1 | 0 | 1 | 1 | 0 |
| 178 | 1 | 1 | 1 | 1 | 1 | 1 | 1 | 0 | 1 | 0 |
| 179 | 1 | 1 | 1 | 0 | 1 | 1 | 1 | 1 | 1 | 0 |
| 180 | 1 | 1 | 0 | 0 | 0 | 1 | 1 | 1 | 1 | 0 |
| 181 | 1 | 1 | 1 | 1 | 1 | 1 | 1 | 1 | 1 | 1 |
| 182 | 1 | 1 | 1 | 0 | 1 | 0 | 0 | 1 | 1 | 0 |
| 183 | 1 | 1 | 1 | 0 | 0 | 1 | 0 | 1 | 1 | 0 |
| 184 | 1 | 1 | 0 | 0 | 0 | 1 | 0 | 1 | 1 | 0 |
| 185 | 1 | 1 | 1 | 0 | 1 | 1 | 0 | 1 | 1 | 0 |
| 186 | 1 | 1 | 0 | 0 | 1 | 1 | 1 | 1 | 1 | 0 |
| 187 | 1 | 1 | 0 | 0 | 1 | 1 | 1 | 1 | 1 | 0 |
| 188 | 1 | 1 | 1 | 0 | 1 | 1 | 0 | 1 | 1 | 0 |
| 189 | 1 | 1 | 0 | 1 | 1 | 0 | 0 | 1 | 1 | 0 |
| 190 | 1 | 1 | 0 | 0 | 1 | 0 | 0 | 1 | 1 | 0 |
| 191 | 1 | 1 | 1 | 0 | 1 | 1 | 0 | 1 | 1 | 0 |
| exclusion percent | 13% | 35% | 26% | 59% | 16% | 10% | 50% | 17% | 1% | |

*Note.* 0 = not eligible, 1 = eligible, 2 = data requested, PA = physical activity, RCT = randomized controlled trial, BMI = body mass index.

1. Abu-Saad, K., Murad, H., Barid, R., Olmer, L., Ziv, A., Younis-Zeidan, N., Kaufman-Shriqui, V., Gillon-Keren, M., Rigler, S., Berchenko, Y., & Kalter-Leibovici, O. (2019). Development and efficacy of an electronic, culturally adapted lifestyle counseling tool for improving diabetes-related dietary knowledge: randomized controlled trial among ethnic minority adults with type 2 diabetes mellitus. Journal of medical Internet research, 21(10), e13674-e13674. https://doi.org/10.2196/13674
2. Adachi, Y., Sato, C., Yamatsu, K., Ito, S., Adachi, K., & Yamagami, T. (2007). A randomized controlled trial on the long-term effects of a 1-month behavioral weight control program assisted by computer tailored advice. Behaviour research and therapy, 45(3), 459-470. https://doi.org/10.1016/j.brat.2006.03.017

3. Adams, M. A., Hurley, J. C., Todd, M., Bhuiyan, N., Jarrett, C. L., Tucker, W. J., Hollingshead, K. E., & Angadi, S. S. (2017). Adaptive goal setting and financial incentives: a 2 × 2 factorial randomized controlled trial to increase adults' physical activity. BMC public health, 17(1), 286-286. https://doi.org/10.1186/s12889-017-4197-8
4. Adams, M. A., Sallis, J. F., Norman, G. J., Hovell, M. F., Hekler, E. B., & Perata, E. (2013). An adaptive physical activity intervention for overweight adults: a randomized controlled trial. PloS one, 8(12), e82901-e82901. https://doi.org/10.1371/journal.pone.0082901
5. Ainscough, K. M., O'Brien, E. C., Lindsay, K. L., Kennelly, M. A., O'Sullivan, E. J., O'Brien, O. A., McCarthy, M., De Vito, G., & McAuliffe, F. M. (2020). Nutrition, behavior change and physical activity outcomes from the PEARS RCT—An mHealth-supported, lifestyle intervention among pregnant women with overweight and obesity. Frontiers in endocrinology, 10. https://doi.org/10.3389/fendo.2019.00938
6. Alharbi, M., Gallagher, R., Kirkness, A., Sibbritt, D., & Tofler, G. (2016). Long-term outcomes from Healthy Eating and Exercise Lifestyle Program for overweight people with heart disease and diabetes. European journal of cardiovascular nursing : journal of the Working Group on Cardiovascular Nursing of the European Society of Cardiology, 15(1), 91-99. https://doi.org/10.1177/1474515114557222
7. Allen, J. K., Stephens, J., Dennison Himmelfarb, C. R., Stewart, K. J., & Hauck, S. (2013). Randomized controlled pilot study testing use of smartphone technology for obesity treatment. Journal of obesity, 2013, 151597-151597. https://doi.org/10.1155/2013/151597
8. Anderson, A. S., Dunlop, J., Gallant, S., Macleod, M., Miedzybrodzka, Z., Mutrie, N., O'Carroll, R. E., Stead, M., Steele, R. J. C., Taylor, R. S., Vinnicombe, S., & Berg, J. (2018). Feasibility study to assess the impact of a lifestyle intervention ('LivingWELL') in people having an assessment of their family history of colorectal or breast cancer. BMJ open, 8(2), e019410-e019410. https://doi.org/10.1136/bmjopen-2017-019410
9. Annesi, J. J., Johnson, P. H., Tennant, G. A., Porter, K. J., & McEwen, K. L. (2016). Weight Loss and the Prevention of Weight Regain: Evaluation of a Treatment Model of Exercise Self-Regulation Generalizing to Controlled Eating. The Permanente journal, 20(3), 15-146. https://doi.org/10.7812/TPP/15-146
10. Annesi, J. J., Unruh-Rewkowski, J. L., & Mareno, N. (2018). Replication and extension of the weight loss for life community-based treatment protocol. behavioral medicine (Washington, D.C.), 44(1), 54-61. https://doi.org/10.1080/08964289.2016.1232241
11. Anton, S. D., LeBlanc, E., Allen, H. R., Karabetian, C., Sacks, F., Bray, G., & Williamson, D. A. (2012). Use of a computerized tracking system to monitor and provide feedback on dietary goals for calorie-restricted diets: the POUNDS LOST study. Journal of diabetes science and technology, 6(5), 1216-1225. https://doi.org/10.1177/193229681200600527
12. Apiñaniz, A., Cobos-Campos, R., Sáez de Lafuente-Moríñigo, A., Parraza, N., Aizpuru, F., Pérez, I., Goicoechea, E., Trápaga, N., & García, L. (2019). Effectiveness of randomized controlled trial of a mobile app to promote healthy lifestyle in obese and overweight patients. Family practice, 36(6), 699-705. https://doi.org/10.1093/fampra/cmz020

13. Astrup, A., Rössner, S., Van Gaal, L., Rissanen, A., Niskanen, L., Al Hakim, M., Madsen, J., Rasmussen, M. F., & Lean, M. E. (2009). Effects of liraglutide in the treatment of obesity: a randomised, double-blind, placebo-controlled study. The Lancet, 374(9701), 1606-1616.
14. Azar, K. M., Koliwad, S., Poon, T., Xiao, L., Lv, N., Griggs, R., & Ma, J. (2016). The electronic cardiometabolic program (ecmp) for patients with cardiometabolic risk: a randomized controlled trial. Journal of medical Internet research, 18(5), e134-e134.
15. Befort, C. A., Klemp, J. R., Austin, H. L., Perri, M. G., Schmitz, K. H., Sullivan, D. K., & Fabian, C. J. (2012). Outcomes of a weight loss intervention among rural breast cancer survivors. Breast cancer research and treatment, 132(2), 631-639. https://doi.org/10.1007/s10549-011-1922-3
16. Beleigoli, A., Andrade, A. Q., Diniz, M. D. F., & Ribeiro, A. L. (2020). Personalized web-based weight loss behavior change program with and without dietitian online coaching for adults with overweight and obesity: randomized controlled trial. Journal of Medical Internet Research, 22(11).
17. Bennell, K., Nelligan, R. K., Schwartz, S., Kasza, J., Alexander, K., Crofts, S. J. C., & Hinman, R. S. (2020). Behavior change text messages for home exercise adherence in knee osteoarthritis: Randomized trial. Journal of Medical Internet Research, 22(9).
18. Bergman, F., Wahlström, V., Stomby, A., Otten, J., Lanthén, E., Renklint, R., Waling, M., Sörlin, A., Boraxbekk, C.-J., Wennberg, P., Öhberg, F., Levine, J. A., & Olsson, T. (2018). Treadmill workstations in office workers who are overweight or obese: a randomised controlled trial. The Lancet. Public health, 3(11), e523-e535. https://doi.org/10.1016/S2468-2667(18)30163-4
19. Berli, C., Stadler, G., Inauen, J., & Scholz, U. (2016). Action control in dyads: A randomized controlled trial to promote physical activity in everyday life. Social science & medicine (1982), 163, 89-97. https://doi.org/10.1016/j.socscimed.2016.07.003
20. Biddle, S. J. H., Edwardson, C. L., Wilmot, E. G., Yates, T., Gorely, T., Bodicoat, D. H., Ashra, N., Khunti, K., Nimmo, M. A., & Davies, M. J. (2015). A Randomised controlled trial to reduce sedentary time in young adults at risk of type 2 diabetes mellitus: project stand (sedentary time and diabetes). PloS one, 10(12), e0143398-e0143398. https://doi.org/10.1371/journal.pone.0143398
21. Braun, A., Portner, J., Grainger, E. M., Hill, E. B., Young, G. S., Clinton, S. K., & Spees, C. K. (2018). Tele-motivational interviewing for cancer survivors: feasibility, preliminary efficacy, and lessons learned. journal of nutrition education and behavior, 50(1), 19-32.e11. https://doi.org/10.1016/j.jneb.2017.05.352
22. Cadmus-Bertram, L., Wang, J. B., Patterson, R. E., Newman, V. A., Parker, B. A., & Pierce, J. P. (2013). Web-based self-monitoring for weight loss among overweight/obese women at increased risk for breast cancer: the HELP pilot study. Psycho-oncology, 22(8), 1821-1828. https://doi.org/10.1002/pon.3219
23. Cadmus-Bertram, L. A., Marcus, B. H., Patterson, R. E., Parker, B. A., & Morey, B. L. (2015). Randomized trial of a fitbit-based physical activity intervention for women. American journal of preventive medicine, 49(3 CC - Gynaecology and Fertility CC - Colorectal), 414-418. https://doi.org/10.1016/j.amepre.2015.01.020
24. Carlson, J. A., Sallis, J. F., Ramirez, E. R., Patrick, K., & Norman, G. J. (2012). Physical activity and dietary behavior change in Internet-based weight loss interventions: comparing two multiple-behavior change indices. Preventive medicine, 54(1), 50-54. https://doi.org/10.1016/j.ypmed.2011.10.018

25. Carr, L. J., Bartee, R. T., Dorozynski, C., Broomfield, J. F., Smith, M. L., & Smith, D. T. (2008). Internet-delivered behavior change program increases physical activity and improves cardiometabolic disease risk factors in sedentary adults: results of a randomized controlled trial. Preventive medicine, 46(5), 431-438. https://doi.org/10.1016/j.ypmed.2007.12.005
26. Carr, L. J., Bartee, R. T., Dorozynski, C. M., Broomfield, J. F., Smith, M. L., & Smith, D. T. (2009). Eight-month follow-up of physical activity and central adiposity: results from an Internet-delivered randomized control trial intervention. Journal of physical activity & health, 6(4), 444-455. https://doi.org/10.1123/jpah.6.4.444
27. Carr, L. J., Karvinen, K., Peavler, M., Smith, R., & Cangelosi, K. (2013). Multicomponent intervention to reduce daily sedentary time: a randomised controlled trial. BMJ open, 3(10). https://doi.org/10.1136/bmjopen-2013-003261
28. Chad-Friedman, E., Pearsall, M., Miller, K. M., Wheeler, A. E., Denninger, J. W., Mehta, D. H., & Dossett, M. L. (2018). Total lifestyle coaching: A pilot study evaluating the effectiveness of a mind–body and nutrition telephone coaching program for obese adults at a community health center. Global Advances In Health and Medicine, 7.
29. Chang, M.-W., Nitzke, S., & Brown, R. (2010). Design and outcomes of a Mothers In Motion behavioral intervention pilot study. Journal of nutrition education and behavior, 42(3 Suppl), S11-21. https://doi.org/10.1016/j.jneb.2010.01.010
30. Conroy, M. B., Yang, K., Elci, O. U., Gabriel, K. P., Styn, M. A., Wang, J., Kriska, A. M., Sereika, S. M., & Burke, L. E. (2011). Physical activity self-monitoring and weight loss: 6-month results of the SMART trial. Medicine and science in sports and exercise, 43(8), 1568-1574. https://doi.org/10.1249/MSS.0b013e31820b9395
31. Coughlin, J. W., Gullion, C. M., Brantley, P. J., Stevens, V. J., Bauck, A., Champagne, C. M., Dalcin, A. T., Funk, K. L., Hollis, J. F., Jerome, G. J., Lien, L. F., Loria, C. M., Myers, V. H., & Appel, L. J. (2013). Behavioral mediators of treatment effects in the weight loss maintenance trial. Annals of Behavioral Medicine, 46(3), 369-381. https://doi.org/10.1007/s12160-013-9517-3
32. Cussler, E. C., Teixeira, P. J., Going, S. B., Houtkooper, L. B., Metcalfe, L. L., Blew, R. M., Ricketts, J. R., Lohman, J. F., Stanford, V. A., & Lohman, T. G. (2008). Maintenance of weight loss in overweight middle-aged women through the Internet. Obesity (Silver Spring, Md.), 16(5), 1052-1060. https://doi.org/10.1038/oby.2008.19
33. Damschroder, L. J., Lutes, L. D., Goodrich, D. E., Gillon, L., & Lowery, J. C. (2010). A small-change approach delivered via telephone promotes weight loss in veterans: results from the ASPIRE-VA pilot study. Patient education and counseling, 79(2), 262-266. https://doi.org/10.1016/j.pec.2009.09.025
34. Darvall, J. N., Wang, A., Nazeem, M. N., Harrison, C. L., Clarke, L., Mendoza, C., Parker, A., Harrap, B., Teale, G., Story, D., & al, e. (2020). A pedometer-guided physical activity intervention for obese pregnant women (the fit mum study): randomized feasibility study. JMIR mhealth and uhealth, 8(5), e15112--e15112-. https://doi.org/10.2196/15112
35. Donnelly, J. E., Goetz, J., Gibson, C., Sullivan, D. K., Lee, R., Smith, B. K., Lambourne, K., Mayo, M. S., Hunt, S., Lee, J. H., Honas, J. J., & Washburn, R. A. (2013). Equivalent weight loss for weight management programs delivered by phone and clinic. Obesity (Silver Spring, Md.), 21(10), 1951-1959. https://doi.org/10.1002/oby.20334

36. Donnelly, J. E., Smith, B. K., Dunn, L., Mayo, M. M., Jacobsen, D. J., Stewart, E. E., Gibson, C., & Sullivan, D. K. (2007). Comparison of a phone vs clinic approach to achieve 10% weight loss. International journal of obesity (2005), 31(8), 1270-1276. https://doi.org/10.1038/sj.ijo.0803568
37. Duncan, M. J., Fenton, S., Brown, W. J., Collins, C. E., Glozier, N., Kolt, G. S., Holliday, E. G., Morgan, P. J., Murawski, B., Plotnikoff, R. C., Rayward, A. T., Stamatakis, E., Vandelanotte, C., & Burrows, T. L. (2020). Efficacy of a multi-component m-health weight-loss intervention in overweight and obese adults: a randomised controlled trial. International journal of environmental research and public health, 17(17). https://doi.org/10.3390/ijerph17176200
38. Eaton, C. B., Hartman, S. J., Perzanowski, E., Pan, G., Roberts, M. B., Risica, P. M., Gans, K. M., Jakicic, J. M., & Marcus, B. H. (2016). A randomized clinical trial of a tailored lifestyle intervention for obese, sedentary, primary care patients. Annals of family medicine, 14(4), 311-319. https://doi.org/10.1370/afm.1952
39. Fazzino, T. L., Fabian, C., & Befort, C. A. (2017). Change in physical activity during a weight management intervention for breast cancer survivors: association with weight outcomes. Obesity (Silver Spring, Md.), 25 Suppl 2(Suppl 2), S109-S115. https://doi.org/10.1002/oby.22007
40. Fukuoka, Y., Gay, C. L., Joiner, K. L., & Vittinghoff, E. (2015). A novel diabetes prevention intervention using a mobile app: a randomized controlled trial with overweight adults at risk. American journal of preventive medicine, 49(2), 223-237. https://doi.org/10.1016/j.amepre.2015.01.003
41. Fukuoka, Y., Vittinghoff, E., & Hooper, J. (2018). A weight loss intervention using a commercial mobile application in latino americans-adelgaza trial. Circulation, 137.
42. Garcia, D. O., Jakicic, J. M., Davis, K. K., Gibbs, B. B., Burke, L. E., & Rickman, A. D. (2014). A pilot feasibility study of a campaign intervention for weight loss among overweight and obese adults. Californian Journal of Health Promotion, 12(3), 56-70. https://doi.org/10.32398/cjhp.v12i3.1581
43. Gerber, B. S., Schiffer, L., Brown, A. A., Berbaum, M. L., Rimmer, J. H., Braunschweig, C. L., & Fitzgibbon, M. L. (2013). Video telehealth for weight maintenance of African-American women. Journal of telemedicine and telecare, 19(5), 266-272. https://doi.org/10.1177/1357633X13490901
44. Giacobbi, P., Jr., Long, D., Nolan, R., Shawley, S., Johnson, K., & Misra, R. (2018). Guided imagery targeting exercise, food cravings, and stress: a multi-modal randomized feasibility trial. Journal of behavioral medicine, 41(1), 87-98. https://doi.org/10.1007/s10865-017-9876-5
45. Grey, E. B., Thompson, D., & Gillison, F. B (2019). Effects of a web-based, evolutionary mismatch-framed intervention targeting physical activity and diet: a randomised controlled trial. International journal of behavioral medicine, 26(6), 645-657. https://doi.org/10.1007/s12529-019-09821-3
46. Groeneveld, I. F., Proper, K. I., van der Beek, A. J., Hildebrandt, V. H., & van Mechelen, W. (2011). Short and long term effects of a lifestyle intervention for construction workers at risk for cardiovascular disease: a randomized controlled trial. BMC public health, 11, 836-836. https://doi.org/10.1186/1471-2458-11-836

47. Haggerty, A. F., Hagemann, A., Barnett, M., Thornquist, M., Neuhouser, M. L., Horowitz, N., Colditz, G. A., Sarwer, D. B., Ko, E. M., & Allison, K. C. (2017). A randomized, controlled, multicenter study of technology-based weight loss interventions among endometrial cancer survivors. Obesity (Silver Spring, Md.), 25 Suppl 2(Suppl 2), S102-S108. https://doi.org/10.1002/oby.22021
48. Hartman, S. J., Nelson, S. H., Cadmus-Bertram, L. A., Patterson, R. E., Parker, B. A., & Pierce, J. P. (2016). Technology- and phone-based weight loss intervention: pilot rct in women at elevated breast cancer risk. American Journal of Preventive Medicine, 51(5), 714-721. https://doi.org/10.1016/j.amepre.2016.06.024
49. Harvey-Berino, J., Pintauro, S., Buzzell, P., DiGiulio, M., Gold, B. C., Moldovan, C., & Ramirez, E. (2002). Does using the Internet facilitate the maintenance of weight loss? International journal of obesity, 26(9 CC - Metabolic and Endocrine Disorders), 1254-1260. https://doi.org/10.1038/sj.ijo.0802051
50. Hellerstedt, W. L., & Jeffery, R. W. (1997). The effects of a telephone-based intervention on weight loss. American journal of health promotion : AJHP, 11(3), 177-182. https://doi.org/10.4278/0890-1171-11.3.177
51. Hernandez-Reyes, A., Camara-Martos, F., Molina Recio, G., Molina-Luque, R., Romero-Saldana, M., & Moreno Rojas, R. (2020). Push Notifications From a Mobile App to Improve the Body Composition of Overweight or Obese Women: randomized Controlled Trial. JMIR mhealth and uhealth, 8(2), e13747--e13747-. https://doi.org/10.2196/13747
52. Hernandez-Reyes, A., Camara-Martos, F., Molina-Luque, R., & Moreno-Rojas, R. (2020). Effect of an mhealth intervention using a pedometer app with full in-person counseling on body composition of overweight adults: randomized controlled weight loss trial. JMIR mhealth and uhealth, 8(5), e16999--e16999-. https://doi.org/10.2196/16999
53. Hersey, J. C., Khavjou, O., Strange, L. B., Atkinson, R. L., Blair, S. N., Campbell, S., Hobbs, C. L., Kelly, B., Fitzgerald, T. M., Kish-Doto, J., Koch, M. A., Munoz, B., Peele, E., Stockdale, J., Augustine, C., Mitchell, G., Arday, D., Kugler, J., Dorn, P., Ellzy, J., Julian, R., Grissom, J., & Britt, M. (2012). The efficacy and cost-effectiveness of a community weight management intervention: a randomized controlled trial of the health weight management demonstration. Preventive medicine, 54(1), 42-49. https://doi.org/10.1016/j.ypmed.2011.09.018
54. Ho, C., Rc, H., Ce, F., So, M., Kitzman-Ulrich, H., & Wj, W. (2011). Computerized self-monitoring and technology-assisted feedback for weight loss with and without an enhanced behavioral component. Patient Education & Counseling, 85(3), 375-382.
55. Hu, G., Tian, H., Zhang, F., Liu, H., Zhang, C., Zhang, S., Wang, L., Liu, G., Yu, Z., Yang, X., Qi, L., Zhang, C., Wang, H., Li, M., Leng, J., Li, Y., Dong, L., & Tuomilehto, J. (2012). Tianjin gestational diabetes mellitus prevention program: study design, methods, and 1-year interim report on the feasibility of lifestyle intervention program. Diabetes research and clinical practice, 98(3), 508-517. https://doi.org/10.1016/j.diabres.2012.09.015
56. Huber, J. M., Shapiro, J. S., Wieland, M. L., Croghan, I. T., Vickers Douglas, K. S., Schroeder, D. R., Hathaway, J. C., & Ebbert, J. O. (2015). Telecoaching plus a portion control plate for weight care management: a randomized trial. Trials, 16, 323-323. https://doi.org/10.1186/s13063-015-0880-1

57. Hutchesson, M. J., Tan, C. Y., Morgan, P., Callister, R., & Collins, C. (2016). Enhancement of self-monitoring in a web-based weight loss program by extra individualized feedback and reminders: randomized trial. Journal of medical Internet research, 18(4), e82-e82. https://doi.org/10.2196/jmir.4100
58. Höchsmann, C., Müller, O., Ambühl, M., Klenk, C., Königstein, K., Infanger, D., Walz, S. P., & Schmidt-Trucksäss, A. (2019). Novel smartphone game improves physical activity behavior in type 2 diabetes. American journal of preventive medicine, 57(1), 41-50. https://doi.org/10.1016/j.amepre.2019.02.017
59. Jakicic, J. M., Davis, K. K., Rogers, R. J., King, W. C., Marcus, M. D., Helsel, D., Rickman, A. D., Wahed, A. S., & Belle, S. H. (2016). Effect of wearable technology combined with a lifestyle intervention on long-term weight loss: the idea randomized clinical trial. JAMA, 316(11), 1161-1171. https://doi.org/10.1001/jama.2016.12858
60. Jakicic, J. M., Marcus, B. H., Lang, W., & Janney, C. (2008). Effect of exercise on 24-month weight loss maintenance in overweight women. Archives of internal medicine, 168(14), 1550-1560. https://doi.org/10.1001/archinte.168.14.1550
61. Jakicic, J. M., Otto, A. D., Lang, W., Semler, L., Winters, C., Polzien, K., & Mohr, K. I. (2011). The effect of physical activity on 18-month weight change in overweight adults. Obesity (Silver Spring, Md.), 19(1), 100-109. https://doi.org/10.1038/oby.2010.122
62. Johnson, K. E., Alencar, M. K., Coakley, K. E., Swift, D. L., Cole, N. H., Mermier, C. M., Kravitz, L., Amorim, F. T., & Gibson, A. L. (2019). Telemedicine-based health coaching is effective for inducing weight loss and improving metabolic markers. Telemedicine journal and e-health: the official journal of the American Telemedicine Association, 25(2), 85-92. https://doi.org/10.1089/tmj.2018.0002
63. Johnson, S. S., Paiva, A. L., Cummins, C. O., Johnson, J. L., Dyment, S. J., Wright, J. A., Prochaska, J. O., Prochaska, J. M., & Sherman, K. (2008). Transtheoretical model-based multiple behavior intervention for weight management: effectiveness on a population basis. Preventive medicine, 46(3), 238-246. https://doi.org/10.1016/j.ypmed.2007.09.010
64. Joseph, R. P., Pekmezi, D., Dutton, G. R., Cherrington, A. L., Kim, Y.-I., Allison, J. J., & Durant, N. H. (2016). Results of a culturally adapted internet-enhanced physical activity pilot intervention for overweight and obese young adult african american women. Journal of transcultural nursing : official journal of the Transcultural Nursing Society, 27(2), 136-146. https://doi.org/10.1177/1043659614539176
65. Kandula, N. R., Dave, S., De Chavez, P. J., Bharucha, H., Patel, Y., Seguil, P., Kumar, S., Baker, D. W., Spring, B., & Siddique, J. (2015). Translating a heart disease lifestyle intervention into the community: the South Asian Heart Lifestyle Intervention (SAHELI) study; a randomized control trial. BMC public health, 15, 1064-1064. https://doi.org/10.1186/s12889-015-2401-2
66. Keyserling, T. C., Samuel-Hodge, C. D., Pitts, S. J., Garcia, B. A., Johnston, L. F., Gizlice, Z., Miller, C. L., Braxton, D. F., Evenson, K. R., Smith, J. C., Davis, G. B., Quenum, E. L., Elliott, N. T. M., Gross, M. D., Donahue, K. E., Halladay, J. R., & Ammerman, A. S. (2016). A community-based lifestyle and weight loss intervention promoting a Mediterranean-style diet pattern evaluated in the stroke belt of North Carolina: the Heart Healthy Lenoir Project. BMC public health, 16, 732-732. https://doi.org/10.1186/s12889-016-3370-9

67. Koniak-Griffin, D., Brecht, M.-L., Takayanagi, S., Villegas, J., Melendrez, M., & Balcazar, H. (2015). A community health worker-led lifestyle behavior intervention for Latina (Hispanic) women: Feasibility and outcomes of a randomized controlled trial. International Journal of Nursing Studies, 52(1), 75-87. https://doi.org/10.1016/j.ijnurstu.2014.09.005
68. Korinek, E. V., Phatak, S. S., Martin, C. A., Freigoun, M. T., Rivera, D. E., Adams, M. A., Klasnja, P., Buman, M. P., & Hekler, E. B. (2018). Adaptive step goals and rewards: a longitudinal growth model of daily steps for a smartphone-based walking intervention. Journal of behavioral medicine, 41(1), 74-86. https://doi.org/10.1007/s10865-017-9878-3
69. L, v. G., P, v. E., Boon, B., Borsboom, G., Visscher, T., & Oenema, A. (2012). Results from an online computer-tailored weight management intervention for overweight adults: randomized controlled trial. Journal of Medical Internet Research, 14(2), e44-e44. https://doi.org/10.2196/jmir.1901
70. Lee, C. H., Cheung, B., Yi, G.-H., Oh, B., & Oh, Y. H. (2018). Mobile health, physical activity, and obesity: Subanalysis of a randomized controlled trial. Medicine, 97(38), e12309-e12309. https://doi.org/10.1097/MD.0000000000012309
71. Lewis, E., Huang, H.-C. C., Hassmén, P., Welvaert, M., & Pumpa, K. L. (2019). Adding telephone and text support to an obesity management program improves behavioral adherence and clinical outcomes. a randomized controlled crossover trial. International journal of behavioral medicine, 26(6), 580-590. https://doi.org/10.1007/s12529-019-09815-1
72. Lison, J. F., Palomar, G., Mensorio, M. S., Banos, R. M., Cebolla-Marti, A., Botella, C., Benavent-Caballer, V., & Rodilla, E. (2020). Impact of a web-based exercise and nutritional education intervention in patients who are obese with hypertension: randomized wait-list controlled trial. Journal of medical Internet research, 22(4), e14196--e14196-. https://doi.org/10.2196/14196
73. Logue, E., Sutton, K., Jarjoura, D., Smucker, W., Baughman, K., & Capers, C. (2005). Transtheoretical model-chronic disease care for obesity in primary care: a randomized trial. Obesity research, 13(5), 917-927. https://doi.org/10.1038/oby.2005.106
74. Lubans, D. R., Morgan, P. J., Collins, C. E., Warren, J. M., & Callister, R. (2009). Exploring the mechanisms of weight loss in the SHED-IT intervention for overweight men: A mediation analysis. International Journal of Behavioral Nutrition and Physical Activity, 6. https://doi.org/10.1186/1479-5868-6-76
75. Lutes, L. D., Daiss, S. R., Barger, S. D., Read, M., Steinbaugh, E., & Winett, R. A. (2012). Small changes approach promotes initial and continued weight loss with a phone-based follow-up: nine-month outcomes from ASPIRES II. American journal of health promotion : AJHP, 26(4), 235-238. https://doi.org/10.4278/ajhp.090706-QUAN-216
76. Martin, C. K., Miller, A. C., Thomas, D. M., Champagne, C. M., Han, H., & Church, T. (2015). Efficacy of SmartLoss, a smartphone-based weight loss intervention: results from a randomized controlled trial. Obesity (Silver Spring, Md.), 23(5), 935-942. https://doi.org/10.1002/oby.21063
77. McConnon, A., Kirk, S. F., Cockroft, J. E., Harvey, E. L., Greenwood, D. C., Thomas, J. D., Ransley, J. K., & Bojke, L. (2007). The Internet for weight control in an obese sample: results of a randomised controlled trial. BMC health services research, 7, 206-206. https://doi.org/10.1186/1472-6963-7-206

78. McKenzie, D. C., Johnson, R. K., Harvey-Berino, J., & Gold, B. C. (2002). Impact of interviewer's body mass index on underreporting energy intake in overweight and obese women. Obesity research, 10(6), 471-477. https://doi.org/10.1038/oby.2002.65
79. Mensorio, M. S., Cebolla-Martí, A., Rodilla, E., Palomar, G., Lisón, J. F., Botella, C., Fernández-Aranda, F., Jimenez-Murcia, S., & Baños, R. M. (2019). Analysis of the efficacy of an internet-based self-administered intervention ("Living Better") to promote healthy habits in a population with obesity and hypertension: An exploratory randomized controlled trial. International journal of medical informatics, 124, 13-23. https://doi.org/10.1016/j.ijmedinf.2018.12.007
80. Monroe, C. M., Geraci, M., Larsen, C. A., & West, D. S. (2019). Feasibility and efficacy of a novel technology-based approach to harness social networks for weight loss: the NETworks pilot randomized controlled trial. Obesity Science and Practice, 5(4), 354-365. https://doi.org/10.1002/osp4.352
81. Morgan, P. J., Callister, R., Collins, C. E., Plotnikoff, R. C., Young, M. D., Berry, N., McElduff, P., Burrows, T., Aguiar, E., & Saunders, K. L. (2013). The SHED-IT community trial: a randomized controlled trial of internet- and paper-based weight loss programs tailored for overweight and obese men. Annals of behavioral medicine : a publication of the Society of Behavioral Medicine, 45(2), 139-152. https://doi.org/10.1007/s12160-012-9424-z
82. Morgan, P. J., Collins, C. E., Plotnikoff, R. C., Cook, A. T., Berthon, B., Mitchell, S., & Callister, R. (2011). Efficacy of a workplace-based weight loss program for overweight male shift workers: the Workplace POWER (Preventing Obesity Without Eating like a Rabbit) randomized controlled trial. Preventive medicine, 52(5), 317-325. https://doi.org/10.1016/j.ypmed.2011.01.031
83. Morgan, P. J., Lubans, D. R., Collins, C. E., Warren, J. M., & Callister, R. (2009). The SHED-IT randomized controlled trial: evaluation of an Internet-based weight-loss program for men. Obesity (Silver Spring, Md.), 17(11), 2025-2032. https://doi.org/10.1038/oby.2009.85
84. Morgan, P. J., Scott, H. A., Young, M. D., Plotnikoff, R. C., Collins, C. E., & Callister, R. (2014). Associations between program outcomes and adherence to Social Cognitive Theory tasks: process evaluation of the SHED-IT community weight loss trial for men. International journal of behavioral nutrition and physical activity, 11(1) (no pagination). https://doi.org/10.1186/s12966-014-0089-9
85. Naami Nazari, L. R. M. T. R., & Javadzade, H. (2020). The effect of web-based educational intervention on physical activity-related energy expenditure among middle-aged women with overweight and obesity: an application of social cognitive theory. Obesity medicine, 18. https://doi.org/10.1016/j.obmed.2020.100181
86. Nakata, Y., Sasai, H., Tsujimoto, T., Hashimoto, K., & Kobayashi, H. (2019). Web-based intervention to promote weight-loss maintenance using an activity monitor: A randomized controlled trial. Preventive Medicine Reports, 14. https://doi.org/10.1016/j.pmedr.2019.100839

87. Navarro, J., Cebolla, A., Llorens, R., Borrego, A., & Baños, R. M. (2020). Manipulating self-avatar body dimensions in virtual worlds to complement an internet-delivered intervention to increase physical activity in overweight women. International journal of environmental research and public health, 17(11). https://doi.org/10.3390/ijerph17114045
88. Nyenhuis, S. M., Balbim, G., Cooley, C., Kim, H., Kitsiou, S., Marquez, D., Wilbur, J., & Sharp, L. (2020). Daily physical activity of urban African American women with asthma. American Journal of Respiratory and Critical Care Medicine, 201(1).
89. Park, M.-J., & Kim, H.-S. (2012). Evaluation of mobile phone and Internet intervention on waist circumference and blood pressure in post-menopausal women with abdominal obesity. International journal of medical informatics, 81(6), 388-394. https://doi.org/10.1016/j.ijmedinf.2011.12.011
90. Patel, M. S., Small, D. S., Harrison, J. D., Fortunato, M. P., Oon, A. L., Rareshide, C. A. L., Reh, G., Szwartz, G., Guszcza, J., Steier, D., & al, e. (2019). Effectiveness of Behaviorally Designed Gamification Interventions with Social Incentives for Increasing Physical Activity among Overweight and Obese Adults Across the United States: the STEP UP Randomized Clinical Trial. JAMA internal medicine. https://doi.org/10.1001/jamainternmed.2019.3505
91. Patrick, K., Calfas, K. J., Norman, G. J., Rosenberg, D., Zabinski, M. F., Sallis, J. F., Rock, C. L., & Dillon, L. W. (2011). Outcomes of a 12-month web-based intervention for overweight and obese men. Annals of behavioral medicine : a publication of the Society of Behavioral Medicine, 42(3), 391-401. https://doi.org/10.1007/s12160-011-9296-7
92. Pellegrini, C. A., Verba, S. D., Otto, A. D., Helsel, D. L., Davis, K. K., & Jakicic, J. M. (2012). The comparison of a technology-based system and an in-person behavioral weight loss intervention. Obesity (Silver Spring, Md.), 20(2), 356-363. https://doi.org/10.1038/oby.2011.13
93. Petrella, R. J., Gill, D. P., Zou, G., De Cruz, A., Riggin, B., Bartol, C., Danylchuk, K., Hunt, K., Wyke, S., Gray, C. M., Bunn, C., & Zwarenstein, M. (2017). Hockey Fans in Training: A Pilot Pragmatic Randomized Controlled Trial. Medicine and science in sports and exercise, 49(12), 2506-2516. https://doi.org/10.1249/MSS.0000000000001380
94. Polzien, K. M., Jakicic, J. M., Tate, D. F., & Otto, A. D. (2007). The efficacy of a technology-based system in a short-term behavioral weight loss intervention. Obesity (Silver Spring, Md.), 15(4), 825-830. https://doi.org/10.1038/oby.2007.584
95. Puhkala, J., Kukkonen-Harjula, K., Aittasalo, M., Mansikkamäki, K., Partinen, M., Hublin, C., Kärmeniemi, P., Sallinen, M., Olkkonen, S., Tokola, K., Ojala, A., Nygård, C. H., & Fogelholm, M. (2016). Lifestyle counseling in overweight truck and bus drivers - Effects on dietary patterns and physical activity. Preventive Medicine Reports, 4, 435-440. https://doi.org/10.1016/j.pmedr.2016.08.012
96. Pullen, C. H., Hageman, P. A., Boeckner, L., Walker, S. N., & Oberdorfer, M. K. (2008). Feasibility of Internet-delivered weight loss interventions among rural women ages 50-69. Journal of geriatric physical therapy (2001), 31(3), 105-112. https://doi.org/10.1519/00139143-200831030-00006
97. Richardson, C. R., Brown, B. B., Foley, S., Dial, K. S., & Lowery, J. C. (2005). Feasibility of adding enhanced pedometer feedback to nutritional counseling for weight loss. Journal of medical Internet research, 7(5), e56-e56. https://doi.org/10.2196/jmir.7.5.e56

98. Rimmer, J. H., Rauworth, A., Wang, E., Heckerling, P. S., & Gerber, B. S. (2009). A randomized controlled trial to increase physical activity and reduce obesity in a predominantly African American group of women with mobility disabilities and severe obesity. Preventive medicine, 48(5), 473-479. https://doi.org/10.1016/j.ypmed.2009.02.008
99. Rock, C. L., Flatt, S. W., Byers, T. E., Colditz, G. A., Demark-Wahnefried, W., Ganz, P. A., Wolin, K. Y., Elias, A., Krontiras, H., Liu, J., Naughton, M., Pakiz, B., Parker, B. A., Sedjo, R. L., & Wyatt, H. (2015). Results of the exercise and nutrition to enhance recovery and good health for you (energy) trial: a behavioral weight loss intervention in overweight or obese breast cancer survivors. Journal of clinical oncology : official journal of the American Society of Clinical Oncology, 33(28), 3169-3176. https://doi.org/10.1200/JCO.2015.61.1095
100. Roesch, S. C., Norman, G. J., Villodas, F., Sallis, J. F., & Patrick, K. (2010). Intervention-mediated effects for adult physical activity: A latent growth curve analysis. Social Science and Medicine, 71(3), 494-501. https://doi.org/10.1016/j.socscimed.2010.04.032
101. Rogers, R. J., Lang, W., Barone Gibbs, B., Davis, K. K., Burke, L. E., Kovacs, S. J., Portzer, L. A., & Jakicic, J. M. (2016). Applying a technology-based system for weight loss in adults with obesity. Obesity Science and Practice, 2(1), 3-12. https://doi.org/10.1002/osp4.18
102. Rollo, M. E., Baldwin, J. N., Hutchesson, M., Aguiar, E. J., Wynne, K., Young, A., Callister, R., Haslam, R., & Collins, C. E. (2020). The feasibility and preliminary efficacy of an ehealth lifestyle program in women with recent gestational diabetes mellitus: A pilot study. International Journal of Environmental Research and Public Health, 17(19), 1-24. https://doi.org/10.3390/ijerph17197115
103. Shapiro, J. R., Koro, T., Doran, N., Thompson, S., Sallis, J. F., Calfas, K., & Patrick, K. (2012). Text4Diet: a randomized controlled study using text messaging for weight loss behaviors. Preventive medicine, 55(5), 412-417. https://doi.org/10.1016/j.ypmed.2012.08.011
104. Sharit, J., Idrees, T., Andrade, A. D., Anam, R., Karanam, C., Valencia, W., Florez, H., & Ruiz, J. G. (2018). Use of an online personal health record's Track Health function to promote positive lifestyle behaviors in Veterans with prediabetes. Journal of health psychology, 23(5), 681-690. https://doi.org/10.1177/1359105316681065
105. Sheppard, V. B., Hicks, J., Makambi, K., Hurtado-de-Mendoza, A., Demark-Wahnefried, W., & Adams-Campbell, L. (2016). The feasibility and acceptability of a diet and exercise trial in overweight and obese black breast cancer survivors: The Stepping STONE study. Contemporary clinical trials, 46, 106-113. https://doi.org/10.1016/j.cct.2015.12.005
106. Simpson, S. A., McNamara, R., Shaw, C., Kelson, M., Moriarty, Y., Randell, E., Cohen, D., Alam, M. F., Copeland, L., Duncan, D., Espinasse, A., Gillespie, D., Hill, A., Owen-Jones, E., Tapper, K., Townson, J., Williams, S., & Hood, K. (2015). A feasibility randomised controlled trial of a motivational interviewing-based intervention for weight loss maintenance in adults. Health technology assessment (Winchester, England), 19(50), v-vi, xix-xxv, 1-378. https://doi.org/10.3310/hta19500

107. Smith, D. T., Carr, L. J., Dorozynski, C., & Gomashe, C. (2009). Internet-delivered lifestyle physical activity intervention: limited inflammation and antioxidant capacity efficacy in overweight adults. Journal of applied physiology (Bethesda, Md. : 1985), 106(1), 49-56. https://doi.org/10.1152/japplphysiol.90557.2008

108. Sniehotta, F. F., Evans, E. H., Sainsbury, K., Adamson, A., Batterham, A., Becker, F., Brown, H., Dombrowski, S. U., Jackson, D., Howell, D., Ladha, K., McColl, E., Olivier, P., Rothman, A. J., Steel, A., Vale, L., Vieira, R., White, M., Wright, P., & Araújo-Soares, V. (2019). Behavioural intervention for weight loss maintenance versus standard weight advice in adults with obesity: A randomised controlled trial in the UK (NULevel Trial). PLoS medicine, 16(5), e1002793-e1002793. https://doi.org/10.1371/journal.pmed.1002793

109. Steeves, J. A., Bassett, D. R., Fitzhugh, E. C., Raynor, H. A., & Thompson, D. L. (2012). Can sedentary behavior be made more active? A randomized pilot study of TV commercial stepping versus walking. The international journal of behavioral nutrition and physical activity, 9, 95-95. https://doi.org/10.1186/1479-5868-9-95

110. Steinberg, D. M., Bennett, G. G., Askew, S., & Tate, D. F. (2015). Weighing every day matters: daily weighing improves weight loss and adoption of weight control behaviors. Journal of the academy of nutrition and dietetics, 115(4), 511-518. https://doi.org/10.1016/j.jand.2014.12.011

111. Steinberg, D. M., Tate, D. F., Bennett, G. G., Ennett, S., Samuel-Hodge, C., & Ward, D. S. (2013). The efficacy of a daily self-weighing weight loss intervention using smart scales and e-mail. Obesity (Silver Spring, Md.), 21(9), 1789-1797. https://doi.org/10.1002/oby.20396

112. Ströbl, V., Knisel, W., Landgraf, U., & Faller, H. (2013). A combined planning and telephone aftercare intervention for obese patients: effects on physical activity and body weight after one year. Journal of rehabilitation medicine, 45(2), 198-205. https://doi.org/10.2340/16501977-1095

113. Swoboda, C. M., Miller, C. K., & Wills, C. E. (2016). Setting Single or Multiple Goals for Diet and Physical Activity Behaviors Improves Cardiovascular Disease Risk Factors in Adults With Type 2 Diabetes: A Pragmatic Pilot Randomized Trial. The Diabetes educator, 42(4), 429-443. https://doi.org/10.1177/0145721716650043

114. Szmeja, M. A., Cramp, C., Grivell, R. M., Deussen, A. R., Yelland, L. N., & Dodd, J. M. (2014). Use of a DVD to provide dietary and lifestyle information to pregnant women who are overweight or obese: a nested randomised trial. BMC pregnancy and childbirth, 14, 409-409. https://doi.org/10.1186/s12884-014-0409-8

115. Tapsell, L. C., Lonergan, M., Batterham, M. J., Neale, E. P., Martin, A., Thorne, R., Deane, F., & Peoples, G. (2017). Effect of interdisciplinary care on weight loss: a randomised controlled trial. BMJ open, 7(7), e014533-e014533. https://doi.org/10.1136/bmjopen-2016-014533

116. Thomas, D., Vydelingum, V., & Lawrence, J. (2011). E-mail contact as an effective strategy in the maintenance of weight loss in adults. Journal of human nutrition and dietetics : the official journal of the British Dietetic Association, 24(1), 32-38. https://doi.org/10.1111/j.1365-277X.2010.01123.x

117. Thomas, J. G., Raynor, H. A., Bond, D. S., Luke, A. K., Cardoso, C. C., Foster, G. D., & Wing, R. R. (2017). Weight loss in Weight Watchers Online with and without an activity tracking device compared to control: A randomized trial. Obesity (Silver Spring, Md.), 25(6), 1014-1021. https://doi.org/10.1002/oby.21846
118. Thorndike, A. N., Sonnenberg, L., Healey, E., Myint-U, K., Kvedar, J. C., & Regan, S. (2012). Prevention of weight gain following a worksite nutrition and exercise program: a randomized controlled trial. American journal of preventive medicine, 43(1), 27-33. https://doi.org/10.1016/j.amepre.2012.02.029
119. Touger-Decker, R., Denmark, R., Bruno, M., O'Sullivan-Maillet, J., & Lasser, N. (2010). Workplace weight loss program; comparing live and internet methods. Journal of occupational and environmental medicine, 52(11), 1112-1118. https://doi.org/10.1097/JOM.0b013e3181f9ee8c
120. Turner-McGrievy, G. M., Beets, M. W., Moore, J. B., Kaczynski, A. T., Barr-Anderson, D. J., & Tate, D. F. (2013). Comparison of traditional versus mobile app self-monitoring of physical activity and dietary intake among overweight adults participating in an mHealth weight loss program. Journal of the American Medical Informatics Association : JAMIA, 20(3), 513-518. https://doi.org/10.1136/amiajnl-2012-001510
121. Turner-McGrievy, G. M., Campbell, M. K., Tate, D. F., Truesdale, K. P., Bowling, J. M., & Crosby, L. (2009). Pounds Off Digitally study: a randomized podcasting weight-loss intervention. American journal of preventive medicine, 37(4), 263-269. https://doi.org/10.1016/j.amepre.2009.06.010
122. Turner-McGrievy, G. M., Davidson, C. R., & Wilcox, S. (2014). Does the type of weight loss diet affect who participates in a behavioral weight loss intervention? A comparison of participants for a plant-based diet versus a standard diet trial. Appetite, 73, 156-162. https://doi.org/10.1016/j.appet.2013.11.008
123. Turner-McGrievy, G. M., Wilcox, S., Boutté, A., Hutto, B. E., Singletary, C., Muth, E. R., & Hoover, A. W. (2017). The dietary intervention to enhance tracking with mobile devices (diet mobile) study: a 6-month randomized weight loss trial. Obesity (Silver Spring, Md.), 25(8), 1336-1342. https://doi.org/10.1002/oby.21889
124. Unick, J. L., Jakicic, J. M., & Marcus, B. H. (2010). Contribution of behavior intervention components to 24-month weight loss. Medicine and science in sports and exercise, 42(4), 745-753. https://doi.org/10.1249/MSS.0b013e3181bd1a57
125. Van Horn, L., Peaceman, A., Kwasny, M., Vincent, E., Fought, A., Josefson, J., Spring, B., Neff, L. M., & Gernhofer, N. (2018). Dietary Approaches to Stop Hypertension Diet and Activity to Limit Gestational Weight: Maternal Offspring Metabolics Family Intervention Trial, a Technology Enhanced Randomized Trial. American journal of preventive medicine, 55(5), 603-614. https://doi.org/10.1016/j.amepre.2018.06.015
126. van Wier, M. F., Ariëns, G. A. M., Dekkers, J. C., Hendriksen, I. J. M., Smid, T., & van Mechelen, W. (2009). Phone and e-mail counselling are effective for weight management in an overweight working population: a randomized controlled trial. BMC public health, 9, 6-6. https://doi.org/10.1186/1471-2458-9-6
127. VanWormer, J. J., Martinez, A. M., Cosentino, D., & Pronk, N. P. (2010). Satisfaction with a weight loss program: what matters? American journal of health promotion : AJHP, 24(4), 238-245. https://doi.org/10.4278/ajhp.080613-QUAN-92

128. Voils, C. I., Adler, R., Strawbridge, E., Grubber, J., Allen, K. D., Olsen, M. K., McVay, M. A., Raghavan, S., Raffa, S. D., & Funk, L. M. (2020). Early-phase study of a telephone-based intervention to reduce weight regain among bariatric surgery patients. Health Psychology, 39(5), 391-402. https://doi.org/http://dx.doi.org/10.1037/hea0000835
129. Voils, C. I., Olsen, M. K., Gierisch, J. M., McVay, M. A., Grubber, J. M., Gaillard, L., Bolton, J., Maciejewski, M. L., Strawbridge, E., & Yancy, W. S., Jr. (2017). Maintenance of weight loss after initiation of nutrition training: a randomized trial. Annals of internal medicine, 166(7), 463-471. https://doi.org/10.7326/M16-2160
130. Watson, A., Bickmore, T., Cange, A., Kulshreshtha, A., & Kvedar, J. (2012). An internet-based virtual coach to promote physical activity adherence in overweight adults: randomized controlled trial. Journal of medical Internet research, 14(1), e1-e1. https://doi.org/10.2196/jmir.1629
131. Watson, S., Woodside, J. V., Ware, L. J., Hunter, S. J., McGrath, A., Cardwell, C. R., Appleton, K. M., Young, I. S., & McKinley, M. C. (2015). Effect of a web-based behavior change program on weight loss and cardiovascular risk factors in overweight and obese adults at high risk of developing cardiovascular disease: randomized controlled trial. Journal of medical Internet research, 17(7), e177-e177. https://doi.org/10.2196/jmir.3828
132. West, D. S., Krukowski, R. A., Finkelstein, E. A., Stansbury, M. L., Ogden, D. E., Monroe, C. M., Carpenter, C. A., Naud, S., & Harvey, J. R. (2020). Adding financial incentives to online group-based behavioral weight control: an rct. American journal of preventive medicine. https://doi.org/10.1016/j.amepre.2020.03.015
133. Willcox, J. C., Wilkinson, S. A., Lappas, M., Ball, K., Crawford, D., McCarthy, E. A., Fjeldsoe, B., Whittaker, R., Maddison, R., & Campbell, K. J. (2017). A mobile health intervention promoting healthy gestational weight gain for women entering pregnancy at a high body mass index: the txt4two pilot randomised controlled trial. BJOG : an international journal of obstetrics and gynaecology, 124(11), 1718-1728. https://doi.org/10.1111/1471-0528.14552
134. Williams, A., Lee, H., Kamper, S. J., O'Brien, K. M., Wiggers, J., Wolfenden, L., Yoong, S. L., Hodder, R. K., Robson, E. K., Haskins, R., McAuley, J. H., & Williams, C. M. (2019). Causal mechanisms of a healthy lifestyle intervention for patients with musculoskeletal pain who are overweight or obese. Clinical rehabilitation, 33(6), 1088-1097. https://doi.org/10.1177/0269215519831419
135. Willis, E. A., Szabo-Reed, A. N., Ptomey, L. T., Steger, F. L., Honas, J. J., Al-Hihi, E. M., Lee, R., Lee, J., Oh, Y., Washburn, R. A., & al, e. (2017). Distance learning strategies for weight management utilizing online social networks versus group phone conference call. Obesity science and practice, 3(2), 134-142. https://doi.org/10.1002/osp4.96
136. Winett, R. A., Anderson, E. S., Wojcik, J. R., Winett, S. G., & Bowden, T. (2007). Guide to health: nutrition and physical activity outcomes of a group-randomized trial of an Internet-based intervention in churches. Annals of behavioral medicine : a publication of the Society of Behavioral Medicine, 33(3), 251-261. https://doi.org/10.1007/BF02879907
137. Wing, R. R., Becofsky, K., Wing, E. J., McCaffery, J., Boudreau, M., Evans, E. W., & Unick, J. (2020). Behavioral and cardiovascular effects of a behavioral weight loss program for people living with hiv. AIDS and behavior, 24(4), 1032-1041. https://doi.org/10.1007/s10461-019-02503-x

138. Xu, X., Leahey, T. M., Boguszewski, K., Krupel, K., Mailloux, K. A., & Wing, R. R. (2017). Self-expansion is associated with better adherence and obesity treatment outcomes in adults. Annals of behavioral medicine : a publication of the Society of Behavioral Medicine, 51(1), 13-17. https://doi.org/10.1007/s12160-016-9823-7
139. Yancy, W. S., Jr., Shaw, P. A., Reale, C., Hilbert, V., Yan, J., Zhu, J., Troxel, A. B., Foster, G. D., & Volpp, K. G. (2019). Effect of escalating financial incentive rewards on maintenance of weight loss: a randomized clinical trial. JAMA network open, 2(11), e1914393-e1914393. https://doi.org/10.1001/jamanetworkopen.2019.14393
140. Yancy, W. S., Jr., Shaw, P. A., Wesby, L., Hilbert, V., Yang, L., Zhu, J., Troxel, A., Huffman, D., Foster, G. D., Wojtanowski, A. C., & Volpp, K. G. (2018). Financial incentive strategies for maintenance of weight loss: results from an internet-based randomized controlled trial. Nutrition & diabetes, 8(1), 33-33. https://doi.org/10.1038/s41387-018-0036-y
141. Yang, Y.-P., Wang, C.-J., Wang, J.-J., Lin, C.-W., Yang, Y.-T. C., Wang, J.-S., Yang, Y. K., & Yang, Y.-C. (2017). The effects of an activity promotion system on active living in overweight subjects with metabolic abnormalities. Obesity research & clinical practice, 11(6), 718-727. https://doi.org/10.1016/j.orcp.2017.06.002
142. Østbye, T., Krause, K. M., Lovelady, C. A., Morey, M. C., Bastian, L. A., Peterson, B. L., Swamy, G. K., Brouwer, R. J. N., & McBride, C. M. (2009). Active Mothers Postpartum: a randomized controlled weight-loss intervention trial. American journal of preventive medicine, 37(3), 173-180. https://doi.org/10.1016/j.amepre.2009.05.016
143. Potts, S., Krafft, J., & Levin, M. E. (2022). A pilot randomized controlled trial of acceptance and commitment therapy guided self-help for overweight and obese adults high in weight self-stigma. Behavior Modification, 46(1), 178-201.
144. Rosas, L. G., Lv, N., Xiao, L., Lewis, M. A., Venditti, E. M., Zavella, P., ... & Ma, J. (2020). Effect of a culturally adapted behavioral intervention for Latino adults on weight loss over 2 years: a randomized clinical trial. *JAMA Network Open*, *3*(12), e2027744-e2027744.
145. Rosas, L. G., Lv, N., Xiao, L., Lewis, M. A., Venditti, E. M., Zavella, P., ... & Ma, J. (2020). Effect of a culturally adapted behavioral intervention for Latino adults on weight loss over 2 years: a randomized clinical trial. JAMA Network Open, 3(12), e2027744-e2027744.
146. Lim, S. L., Ong, K. W., Johal, J., Han, C. Y., Yap, Q. V., Chan, Y. H., ... & Khoo, C. M. (2021). Effect of a smartphone app on weight change and metabolic outcomes in Asian adults with type 2 diabetes: a randomized clinical trial. JAMA network open, 4(6), e2112417-e2112417.
147. Lugones-Sanchez, C., Recio-Rodriguez, J. I., Agudo-Conde, C., Repiso-Gento, I., G Adalia, E., Ramirez-Manent, J. I., ... & EVIDENT 3 Investigators. (2022). Long-term effectiveness of a smartphone app combined with a smart band on weight loss, physical activity, and caloric intake in a population with overweight and obesity (evident 3 study): randomized controlled trial. Journal of medical Internet research, 24(2), e30416.
148. Levin, M. E., Petersen, J. M., Durward, C., Bingeman, B., Davis, E., Nelson, C., & Cromwell, S. (2021). A randomized controlled trial of online acceptance and commitment therapy to improve diet and physical activity among adults who are overweight/obese. Translational Behavioral Medicine, 11(6), 1216-1225.

149. Taylor, R., Rollo, M. E., Baldwin, J. N., Hutchesson, M., Aguiar, E. J., Wynne, K., ... & Collins, C. E. (2022). Evaluation of a Type 2 diabetes risk reduction online program for women with recent gestational diabetes: a randomised trial. International Journal of Behavioral Nutrition and Physical Activity, 19(1), 35.
150. Downs, D. S., Savage, J. S., Rivera, D. E., Pauley, A. M., Leonard, K. S., Hohman, E. E., ... & Kunselman, A. (2021). Adaptive, behavioral intervention impact on weight gain, physical activity, energy intake, and motivational determinants: results of a feasibility trial in pregnant women with overweight/obesity. Journal of behavioral medicine, 44(5), 605-621.
151. Stansbury, M. L., Harvey, J., Krukowski, R. A., Pellegrini, C. A., Wang, X., & West, D. S. (2022). Describing transitions in adherence to physical activity self-monitoring and goal attainment in an online behavioral weight loss program: Secondary analysis of a randomized controlled trial. Journal of Medical Internet Research, 24(1), e30673.
152. Ansari, K., Afshari, P., Abedi, P., & Haghighizadeh, M. (2022). Comparing the effects of text messaging and mobile social networking on physical activity and anthropometric indices of middle-aged women: a randomized controlled trial. BMC Women's Health, 22(1), 18.
153. Drew, R. J., Morgan, P. J., & Young, M. D. (2022). Mechanisms of an eHealth program targeting depression in men with overweight or obesity: A randomised trial. Journal of affective disorders, 299, 309-317.
154. Bennell, K. L., Lawford, B. J., Keating, C., Brown, C., Kasza, J., Mackenzie, D., ... & Hinman, R. S. (2022). Comparing video-based, telehealth-delivered exercise and weight loss programs with online education on outcomes of knee osteoarthritis: a randomized trial. Annals of internal medicine, 175(2), 198-209.
155. Burke, L. E., Sereika, S. M., Parmanto, B., Bizhanova, Z., Kariuki, J. K., Cheng, J., ... & Conroy, M. B. (2022). Effect of tailored, daily feedback with lifestyle self-monitoring on weight loss: The SMARTER randomized clinical trial. Obesity, 30(1), 75-84.
156. Black, M., & Brunet, J. (2021). A wearable activity tracker intervention with and without weekly behavioral support emails to promote physical activity among women who are overweight or obese: randomized controlled trial. JMIR mHealth and uHealth, 9(12), e28128.
157. Bughin, F., Bui, G., Ayoub, B., Blervaque, L., Saey, D., Avignon, A., ... & Hayot, M. (2021). Impact of a mobile telerehabilitation solution on metabolic health outcomes and rehabilitation adherence in patients with obesity: randomized controlled trial. JMIR mHealth and uHealth, 9(12), e28242.
158. Hassoon, A., Baig, Y., Naiman, D. Q., Celentano, D. D., Lansey, D., Stearns, V., ... & Appel, L. J. (2021). Randomized trial of two artificial intelligence coaching interventions to increase physical activity in cancer survivors. npj Digital Medicine, 4(1), 168.
159. Francis, S. L., Simmering, J. E., Polgreen, L. A., Evans, N. J., Hosteng, K. R., Carr, L. J., ... & Polgreen, P. M. (2021). Gamifying accelerometer use increases physical activity levels of individuals pre-disposed to type II diabetes. Preventive medicine reports, 23, 101426.

160. Eslami, E., Mohammad Alizadeh Charandabi, S., Farshbaf Khalili, A., Asghari Jafarabadi, M., & Mirghafourvand, M. (2022). The effect of a lifestyle training package on physical activity and nutritional status in obese and overweight pregnant women: A randomized controlled clinical trial. International Journal of Nursing Practice, 28(6), e12992.
161. Taylor, A. H., Taylor, R. S., Ingram, W. M., Anokye, N., Dean, S., Jolly, K., ... & Cavanagh, C. (2020). Adding web-based behavioural support to exercise referral schemes for inactive adults with chronic health conditions: the e-coachER RCT. Health Technology Assessment (Winchester, England), 24(63), 1.
162. Lugones-Sanchez, C., Sanchez-Calavera, M. A., Repiso-Gento, I., Adalia, E. G., Ramirez-Manent, J. I., Agudo-Conde, C., ... & EVIDENT 3 Investigators. (2020). Effectiveness of an mHealth intervention combining a smartphone app and smart band on body composition in an overweight and obese population: randomized controlled trial (EVIDENT 3 study). JMIR mHealth and uHealth, 8(11), e21771.
163. Klasnja, P., Rosenberg, D. E., Zhou, J., Anau, J., Gupta, A., & Arterburn, D. E. (2021). A quality-improvement optimization pilot of BariFit, a mobile health intervention to promote physical activity after bariatric surgery. Translational Behavioral Medicine, 11(2), 530-539.
164. Lewis, Z. H., Ottenbacher, K. J., Fisher, S. R., Jennings, K., Brown, A. F., Swartz, M. C., ... & Lyons, E. J. (2020). Effect of electronic activity monitors and pedometers on health: results from the TAME health pilot randomized pragmatic trial. International journal of environmental research and public health, 17(18), 6800.
165. Bernal-Jiménez, M. Á., Calle, G., Gutiérrez Barrios, A., Gheorghe, L. L., Cruz-Cobo, C., Trujillo-Garrido, N., ... & Santi-Cano, M. J. (2024). Effectiveness of an interactive mhealth app (evite) in improving lifestyle after a coronary event: randomized controlled trial. JMIR mHealth and uHealth, 12, e48756.
166. Bizhanova, Z., Sereika, S. M., Brooks, M. M., Rockette-Wagner, B., Kariuki, J. K., & Burke, L. E. (2023). Identifying predictors of adherence to the physical activity goal: a secondary analysis of the SMARTER weight loss trial. Medicine and science in sports and exercise, 55(5), 856.
167. Bruce, J. M., Cozart, J. S., Shook, R. P., Befort, C., Siengsukon, C. F., Simon, S., ... & Bruce, A. S. (2023). Modifying diet and exercise in multiple sclerosis (MoDEMS): A randomized controlled trial for behavioral weight loss in adults with multiple sclerosis and obesity. Multiple Sclerosis Journal, 29(14), 1860-1871.
168. Cebrick-Grossman, J. A., & Fetherman, D. L. (2024). A Workplace Physical Activity Intervention and a Smartphone App for Overweight and Obese Sedentary Women. Workplace Health & Safety, 72(10), 431-438.
169. Cebrick-Grossman, J. A., & Fetherman, D. L. (2024). A Worksite Intervention Program for Obese Sedentary Women Using Wearable Technology. Workplace Health & Safety, 21650799241254402.
170. Chen, H. H., Lee, C. F., Huang, J. P., Hsiung, Y., & Chi, L. K. (2023). Effectiveness of a nurse-led mHealth app to prevent excessive gestational weight gain among overweight and obese women: A randomized controlled trial. Journal of Nursing Scholarship, 55(1), 304-318.

171. Cheng, J., Costacou, T., Sereika, S. M., Conroy, M. B., Parmanto, B., Rockette-Wagner, B., ... & Burke, L. E. (2023). Effect of an mHealth weight loss intervention on Healthy Eating Index diet quality: the SMARTER randomised controlled trial. British Journal of Nutrition, 130(11), 2013-2021.
172. Emerson, J. A., Schumacher, L. M., Bond, D. S., Thomas, J. G., & Lillis, J. (2023). Physical activity changes during an automated online weight loss program. Journal of Behavioral Medicine, 46(4), 680-688.
173. Encantado, J., Marques, M. M., Gouveia, M. J., Santos, I., Sánchez-Oliva, D., O'Driscoll, R., ... & Palmeira, A. L. (2023). Testing motivational and self-regulatory mechanisms of action on device-measured physical activity in the context of a weight loss maintenance digital intervention: A secondary analysis of the NoHoW trial. Psychology of sport and exercise, 64, 102314.
174. Fichtner, U. A., Armbruster, C., Bischoff, M., Maiwald, P., Sehlbrede, M., Tinsel, I., ... & Farin-Glattacker, E. (2022). Evaluation of an interactive web-based health program for weight loss—a randomized controlled trial. International journal of environmental research and public health, 19(22), 15157.
175. Greco, F., Tarsitano, M. G., Cosco, L. F., Quinzi, F., Folino, K., Spadafora, M., ... & Emerenziani, G. P. (2024). The effects of online home-based pilates combined with diet on body composition in women affected by obesity: a preliminary study. Nutrients, 16(6), 902
176. Hanafiah, A. N., Aagaard-Hansen, J., Cheah, J. C., Norris, S. A., Karim, Z. B., Skau, J. K., ... & Hanson, M. (2022). Effectiveness of a complex, pre-conception intervention to reduce the risk of diabetes by reducing adiposity in young adults in Malaysia: The Jom Mama project–A randomised controlled trial. Journal of global health, 12.
177. Jay, M. R., Wittleder, S., Vandyousefi, S., Illenberger, N., Nicholson, A., Sweat, V., ... & Wylie-Rosett, J. (2024). A cluster-randomized study of technology-assisted health coaching for weight management in primary care. The Annals of Family Medicine, 22(5), 392-399.
178. Joseph, R. P., Todd, M., Ainsworth, B. E., Vega-López, S., Adams, M. A., Hollingshead, K., ... & Keller, C. (2023). Smart Walk: a culturally tailored smartphone-delivered physical activity intervention for cardiometabolic risk reduction among African American women. International journal of environmental research and public health, 20(2), 1000.
179. Khazaei, R., Maleklou, F., Bodaghabadi, Z., Tavana, M. M., Kluzek, S., Sharafi, S. E., ... & Alizadeh, Z. (2024). Developing an 8-week, tele-education weight control and exercise programme, and evaluating its effects on weight and pain reduction in patients with obesity and knee osteoarthritis: a double-blinded randomised clinical trial. Musculoskeletal Care, 22(3), e1926.
180. Kim, G., Kim, S., Lee, Y. B., Jin, S. M., Hur, K. Y., & Kim, J. H. (2024). A randomized controlled trial of an app-based intervention on physical activity and glycemic control in people with type 2 diabetes. BMC medicine, 22(1), 185.
181. Kohl, J., Brame, J., Centner, C., Wurst, R., Fuchs, R., Sehlbrede, M., ... & König, D. (2023). Effects of a web-based lifestyle intervention on weight loss and cardiometabolic risk factors in adults With Overweight and Obesity: randomized controlled clinical trial. Journal of Medical Internet Research, 25, e43426.

182. Kondo, M., Okitsu, T., Waki, K., Yamauchi, T., Nangaku, M., & Ohe, K. (2022). Effect of information and communication technology–based self-management system dialbeticslite on treating abdominal obesity in the specific health guidance in japan: randomized controlled trial. JMIR formative research, 6(3), e33852.
183. Lakka, T. A., Aittola, K., Järvelä-Reijonen, E., Tilles-Tirkkonen, T., Männikkö, R., Lintu, N., ... & Pihlajamäki, J. (2023). Real-world effectiveness of digital and group-based lifestyle interventions as compared with usual care to reduce type 2 diabetes risk–A stop diabetes pragmatic randomised trial. The Lancet Regional Health–Europe, 24.
184. Lambert, J., Taylor, A., Streeter, A., Greaves, C., Ingram, W. M., Dean, S., ... & Campbell, J. (2022). A process evaluation, with mediation analysis, of a web-based intervention to augment primary care exercise referral schemes: the e-coachER randomised controlled trial. International Journal of Behavioral Nutrition and Physical Activity, 19(1), 128.
185. Mueller, J., Richards, R., Jones, R. A., Whittle, F., Woolston, J., Stubbings, M., ... & Ahern, A. L. (2022). Supporting weight management during COVID-19: a randomized controlled trial of a web-based, ACT-based, Guided Self-Help Intervention. Obesity Facts, 15(4), 550-559.
186. Múzquiz-Barberá, P., Ruiz-Cortés, M., Herrero, R., Vara, M. D., Escrivá-Martínez, T., Baños, R. M., ... & Lisón, J. F. (2023). "Own doctor" presence in a web-based lifestyle intervention for adults with obesity and hypertension: A randomized controlled trial. Frontiers in Public Health, 11, 1115711.
187. Ruiz-Cortés, M., Múzquiz-Barberá, P., Herrero, R., Vara, M. D., Escrivá-Martínez, T., Carcelén, R., ... & Lisón, J. F. (2023). How the presence of a doctor known to patients impacts a web-based intervention to promote physical activity and healthy eating behaviour in individuals with an overweight/obesity–hypertension phenotype: a randomised clinical trial. Nutrients, 15(7), 1624.
188. Simmering, J. E., Polgreen, L. A., Francis, S. L., Strom, A. J., Segre, A. M., & Polgreen, P. M. (2024). Using a fitbit-based walking game to improve physical activity among us veterans. Military medicine, usae280.
189. Spring, B., Pfammatter, A. F., Scanlan, L., Daly, E., Reading, J., Battalio, S., ... & Nahum-Shani, I. (2024). an adaptive behavioral intervention for weight loss management: a randomized clinical trial. JAMA.
190. Stansbury, M. L., Krukowski, R. A., You, W., Harvey, J. R., & West, D. S. (2022). Effects of meeting steps-based and minutes-based physical activity goals on weight loss in online behavioral weight control: seemingly unrelated regression analysis. Health Psychology and Behavioral Medicine, 10(1), 956-972.
191. Yıldız, M., & Baysal, H. Y. (2024). The effect of web-based and face-to-face training given to office workers on health beliefs and physical activity levels regarding obesity. International Journal of Nursing Practice, 30(3), e13193.

Table 3s

*Characteristic Summary of Included Studies*

| | Number of Studies (*k*) |
|---|---|
| Sampling Method | |
| Not reported | 1 |
| Probability sampling | 1 |
| Non-probability sampling | 12 |
| Assignment Method | |
| Individualized | 12 |
| Clustered | 2 |
| Matching | |
| Yes | 4 |
| No | 10 |
| Study Location | |
| US | 1 |
| Non-US | 8 |
| Not reported | 5 |
| Risk of Bias | |
| High risk | 3 |
| Some risk | 10 |
| Low risk | 1 |
| Gender | |
| Not reported | 1 |
| Mixed gender | 8 |
| Male only | 1 |
| Female only | 4 |
| Ethnicity | |
| Not reported | 7 |
| Multiple ethnic group | 6 |
| Only black | 1 |
| Weight Status | |
| Overweight (BMI: 25 - 29.9) | 1 |
| Obese (BMI: 30 and above) | 2 |
| Overweight and obese | 11 |
| Intervention Duration | |
| Not reported | 1 |
| Short term (less than 6 months) | 6 |

| | |
|---|---|
| Intermediate term (from 6 months to 12 months) | 7 |
| Long term (more than 12 months) | 0 |
| Theory | |
| Not used | 7 |
| Used | 7 |
| Assessment | |
| Self-reports (including interview with questionnaire) | 10 |
| Accelerometers | 3 |
| Both self-reports and accelerometers | 1 |
